# A systematic review of early motor interventions for infants with congenital heart disease and open-heart surgery

**DOI:** 10.1186/s13643-023-02320-3

**Published:** 2023-08-25

**Authors:** Rahel Kaeslin, Beatrice Latal, Elena Mitteregger

**Affiliations:** 1 Child Development Center, University Children’s Hospital Zurich, 8032 Zurich, Switzerland; 2 University of Zurich, Zurich, Switzerland; 3 Children’s Research Center, University Children’s Hospital Zurich, Zurich, Switzerland

**Keywords:** Congenital heart disease, Open-heart surgery, Infant, Child, Early motor intervention, Motor outcome, Exercise, Physiotherapy, Parental involvement and consulting

## Abstract

**Background:**

Motor development delay is the first neurodevelopmental impairment that becomes apparent in infants with congenital heart disease (CHD). Early interventions have addressed high-risk groups like infants born preterm, but little is known about interventions to improve motor outcome in CHD infants at risk of motor delay. The purpose of this review was to systematically review the literature on type and effect of motor intervention applied during the first year of life in infants with CHD following open-heart surgery.

**Methods:**

Scoping searches were performed in May 2020 and April 2023 via MEDLINE, Embase, CINAHL, Cochrane, PsycINFO, PEDro, and Scopus. The review included studies published in English from 2015 to 2022. Primary outcome was infants’ motor development measured by standardized and non-standardized motor assessments, and if available, infants’ language and cognitive development, and any parental quality-of-life assessments as secondary outcomes. The studies’ quality was evaluated with a modified Newcastle-Ottawa scale.

**Results:**

Four papers with low to high methodological quality met inclusion criteria. All studies investigated the influence of early physiotherapy. Four studies involved parents, and three studies used standardized tools to assess motor outcomes. No conclusion can be drawn about any positive effect of early motor interventions.

**Conclusions:**

Early motor intervention in CHD infants may improve motor development; however, the few existing studies do not provide clear evidence. Thus, more prospective early intervention studies are needed.

**Trial registration:**

PROSPERO CRD42020200981.

**Supplementary Information:**

The online version contains supplementary material available at 10.1186/s13643-023-02320-3.

## Background

Congenital heart disease (CHD) is one of the most common congenital malformations; it occurs in approximately 10 of 1000 liveborn children worldwide and affects millions of newborns [1, 2]. CHD comprises a range of congenital heart defects and is defined as a gross structural abnormality of the heart or intrathoracic great vessels that is actually or possibly of functional significance [3]. Advances in prenatal diagnosis, medical care, and surgical interventions have increased survival rates dramatically to a current rate of around 90%, even for the most severe forms [4]. As survival rates have increased, the research focus has shifted to examining potential neurodevelopmental sequelae. Complex CHD may be associated with an increased risk of abnormal brain development, perioperative brain injury, and consequently neurodevelopmental impairments [5]. Motor development delay is the first domain in which congenital heart disease (CHD) becomes apparent as delayed acquisition of milestones associated with generalized muscular hypotonia [6, 7].

Despite the knowledge that infants with CHD after open-heart surgery are at high risk of neurodevelopmental delay, children with CHD receive fewer therapies than preterm children. This suggests a lack of awareness regarding the challenges infants with CHD are facing [8]. In preterm infants, a population known to be at high risk for neurodevelopmental delay like cerebral palsy (CP) however, early motor interventions have been well investigated. Reviews state that early interventions for preterm infants [9] and infants at risk of CP have a positive influence on motor and cognitive outcomes during infancy [10]. The heterogeneity of studies investigating these programs limits the conclusion about their effectiveness. Nonetheless, literature states that for early intervention to be effective, it should include the following components: start early; be intense, active, and tailored for each individual; should be fun and goal directed; and actively involve the baby and its family [11–13].

Although a substantial number of studies have investigated neuromotor outcomes in children with CHD, little is known about studies aiming to improve neuromotor developmental outcomes for infants with CHD after open-heart surgery. Bolduc et al. [14] highlight in their systematic review that patients with CHD have an increased risk of motor impairments across infancy, childhood, and adolescence. Liamlahi et al. [15] reported that children with CHD perform more poorly in all motor domains at school age than their healthy controls. Likewise, adolescents showed significantly more motor problems in fine and gross motor functions than their controls after open-heart surgery in early childhood [16]. In addition, caregivers, teachers, and medical professionals may be concerned about the underlying cardiac condition [17] and thus may overprotect CHD children and restrict them from physical activities. Furthermore, we have demonstrated that parents’ burdens and needs play a crucial role in promoting their infants’ motor development [18].

These findings were the rationale for a systematic literature review regarding early motor interventions for infants with CHD who have undergone open-heart surgery. Thus, this systematic review aims to summarize the existing literature on motor interventions for infants with CHD after open-heart surgery and investigate the effects of early motor interventions on these children’s neuromotor development.

## Methods

The methods of this systematic review were predefined and summarized in a systematic review protocol adhering to the PRISMA-P guideline [19]. The protocol was registered in the International Prospective Register of Systematic Reviews (PROSPERO) on 07.11.2020 (registration number CRD42020200981) and amended on 02.11.2021.

### Eligibility criteria

#### Population

Infants with all types of CHD, with or without syndromal or genetic abnormalities, and a mean age of less than 12 months at initiation of the intervention were included in this review (Table 1). Prematurity was not an exclusion criterion. To ensure comparability, all infants with CHD had to have had open-heart surgery.

**Table 1:** Eligibility criteria

	**Inclusion criteria**	**Exclusion criteria**
**Age**	Patients with a mean age younger or equal to 1 year at initiation of the intervention	Patients with a mean age older than 1 year at study enrolment
**CHD**	Any congenital heart disease Open-heart surgery Including genetic defects and syndromes	
**Intervention**	Any type of intervention aiming to improve motor developmental outcome	Interventions such as surgeries and the use of new drugs
**Primary outcome**	Motor development outcomes using standardized and non-standardized assessments	
**Secondary outcomes**	Language and cognitive development, parental quality-of-life outcomes if available	
**Study design**	Peer-reviewed quantitative research studies like RCTs and cohort studies	Reviews, research protocols, ongoing studies, case studies, opinions, and comments

#### Types of interventions

Studies were included in this systematic review if they investigated motor interventions that aim to promote infants’ motor development and motor developmental outcome measures, such as physiotherapy, physical therapy, occupational therapy, exercise, movement therapy, motor therapy, and rehabilitation. Interventions could be performed at hospitals, outpatient settings, or at home.

This review excluded trials focusing on interventions such as surgeries and the use of new drugs.

#### Study designs

Original peer-reviewed studies with quantitative design, like RCTs and cohort studies, were included (Table 1). We excluded reviews, research protocols, ongoing studies, case studies, opinions, and comments as this would not allow us to report on the influence of motor interventions on motor outcome.

#### Outcome measures

Infants’ motor development was our primary outcome including standardized and non-standardized motor assessment tools. Motor assessments are considered as standardized if their psychometric properties are reliable and valid, and normative data are available. Assessments with unknown psychometric properties are considered as not standardized. The motor composite score of the Bayley Scales of Infant and Toddler Development (BSID III) [20] including both fine and gross motor skills, and the Alberta Infant Motor Score (AIMS) [21], is widely used standardized assessments in infants with CHD. Assessments with unknown psychometric properties are considered as not standardized.

Secondary outcomes included, if reported, children’s language and cognitive development assessments like the BSID III composite score for language and cognition and parents’ quality of life like the SF36 [22] or Parental Stress Index [23]. We included motor interventions that started in infants’ first year of life regardless of their follow-up time.

### Search strategy and screening

The following electronic databases were searched: MEDLINE, Embase, CINAHL, Cochrane, PsycINFO, PEDro, and Scopus. M. G.-P., a librarian of the University Zurich, developed the search strategy and conducted the search using subject headings (MeSH, EMTREE, and PsycINDEX thesaurus) and free-text words related to CHD and early motor intervention, physiotherapy, physical therapy, occupational therapy, exercise, kinesiotherapy, support, movement therapy, motor therapy, neurodevelopment, treatment outcome, motor outcome assessment, psychomotor disorder, and neurologic disorder. The initial literature search was conducted in May 2020 and included studies from 2015 to 2020. Additionally, we searched reference lists of reviews for relevant literature we could include in February 2021. An updated search was conducted in April 2023 including studies published in 2020–2023. The search protocols are included as Supplemental materials 1 and 2. Studies were only included if the full text was available in English or German.

R. K. and E. M. screened titles and abstracts independently first using the program Covidence [24]. R. K. retrieved full texts for eligibility. R. K. and E. M. read and assessed eligible articles. Remaining uncertainties of studies to be considered for the review were discussed with B. L. Reviewers were not blinded to studies’ authors or authors’ affiliations during the study selection process.

### Data extraction

Study characteristics were extracted by RK and included the following parameters: authors’ name, year of publication, study design, country of origin, participants, surgery, duration of cardiopulmonary bypass and intensive care unit (ICU), length of hospital stay, infants’ mean age, type, frequency, duration and period of intervention, motor outcome measures, infants’ age at the outcome, other outcomes, and parents’ quality of life. We were unable to conduct a meta-analysis as included studies did not provide enough information to evaluate effectiveness. Thus, our systematic review is descriptive.

### Quality assessment

The quality of the studies was evaluated with a modified checklist, which was based on the Newcastle-Ottawa scale for the quality of cohort studies (http://www.ohri.ca/programs/clinical_epidemiology/oxford.asp, accessed August 2021). We adapted the scale so that “ascertainment of exposure” was interpreted as “ascertainment of intervention.” The Newcastle-Ottawa allows transparent rating of the bias in each of the domains assessed. The bias assessment was performed by three independent researchers (R. K., E. M., B. L.). We rated all included studies with this assessment to ensure comparability. Studies were not weighted or excluded based on the bias assessment, but its results were considered in the overall discussion.

## Results

### Selection of studies

**Fig. 1:**
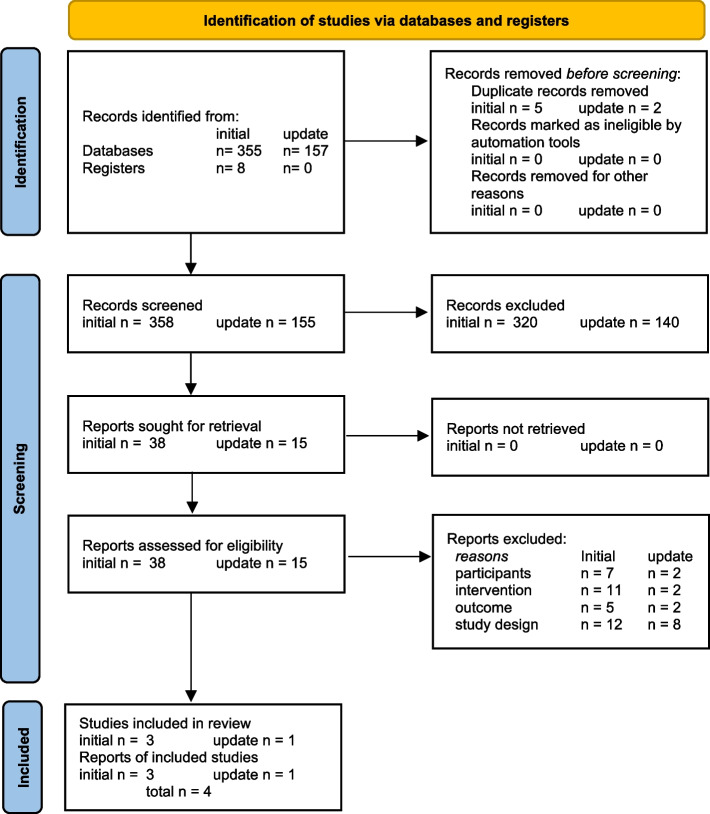
PRISMA 2020 flow diagram for new systematic reviews which included searches of databases and registers only

The initial search 2020 identified a total number of 358 studies after removing five duplications. After title and abstract screening, 38 full-text articles remained, and 35 articles were excluded by the predefined exclusion criteria. Three studies met the inclusion criteria. The update search 2023 identified a total number of 155 studies after removing two duplications. After title and abstract screening, 15 full-text articles remained, and 14 articles were excluded by the predefined exclusion criteria. One article met the inclusion criteria. Finally, four articles were approved for the systematic review by all authors (Fig. 1).

### Study characteristics and quality assessment

Included studies measured motor outcomes in 135 patients in total. All studies were observations of varying qualities prohibiting a meta-analysis. The characteristics and quality assessment of the studies included are summarized in Table 2. The four studies comprised one RCT [25], two retrospective controlled cohort studies [26, 27], and one cohort study [28]. The RCT had a level of evidence of II, the controlled cohort studies a level of III and IV, and the cohort study a level of IV.

Fourdain et al. [26] and Haseba et al. [28] evaluated existing physiotherapy intervention programs and examined factors determining better outcome. Uzark et al. [25] and Long et al. [27] prospectively examined new physical programs. However, the study of Long et al. [27] was stopped due to difficulties in recruitment. Group sizes varied from 6 to 51 children. Three studies [25–27] excluded infants born prematurely. All studies were written in English and published between 2015 and 2022.

**Table 2:** Summary and quality assessment

						**Intervention**					**Outcome**		**Quality assessment**			
**Study** (year)	**Study design**	**Study groups**	**Study site**	***n***	**Mean age at surgery**	**Type**	**Duration**	**Frequency**	**Time period**	**Profession**	**Measure**	**Age**	**Level of evidence**	**Selection**	**Comparability**	**Outcome**
Uzark (2022) [25]	RCT	IG (G1, G2), CG	Michigan, USA	49	5 days	PT	> 5 min	3×/day	3 m	P, PT, N, PSY	AIMS	3 m	II	**★★★★**	☆☆	**★★★**
Fourdain (2020) [26]	CCS	IG (O, R), CG	Montreal, USA	29	10 days	PT	60 min	O: 1–2×	4 m	P, PT	AIMS, BSID III	4 m, 12 m, 24 m	III	**★★★★**	☆☆	**★★★**
								R: 3–6×								
Long (2015) [27]	CCS	IG, CG	Melbourne, Australia	6	6 days	PT	Na	Na	Na	PT	AIMS	3 m, 8 m, 12 m	IV	**★★**☆☆	☆☆	**★**☆☆
Haseba (2018) [28]	rCS	IG (cyn/acyan)	Kagoshima, Japan	51	268.4 days	PT	20–60 min	1–3/day+	Mean 18 days	PT	NGMA	Mean 25 days after surgery	IV	**★★★**☆☆	☆☆	**★**☆☆
								5–6/week								

*acyn* acyanotic group, *AIMS* Alberta Infant Motor Scale, *BSID III* Bayley Scales of Infant Development, *CG* control group, *CCS* controlled cohort study, *rCS* retrospective cohort study, *cyn* cyanotic group, *G1* group 1, *G2* group 2, *IG* intervention group, *m* months, *na* not available, *NGMA* nine-grade mobility assessment scale, *N* nurse, *n* number, *O* occasional group, *P* parents, *PSY* psychologist, *PT* physiotherapist, *R* regular group, *RCT* randomized control trial, *w* weeks

### The studies

Uzark et al. [25] evaluated the feasibility and efficacy of a “tummy time” intervention to improve motor skills in infants after cardiac surgery in the US. They included 64 infants < 4 months of age and who were ≥ 24 h intubated after surgery. Infants with central nervous abnormalities and < 36 weeks’ gestation prior surgery were excluded. The study lasted for 3 months, and infants were randomly assigned into three groups. Parents of the “inpatient intervention group” (*n* = 20), and bedside staff, received tummy time recommendations and instructions of prone positioning at hospital. Prior discharge, parents were instructed to practice tummy time 3× 5 min daily and advance daily prone duration. Parents of the “inpatient plus outpatient intervention group” (*n* = 21) additionally received outpatient telephone calls to support families performing tummy time by a psychologist. Parents of the “control group/standard of care group” (*n* = 23) only received a brochure describing the importance of tummy time during play. Infants’ motor development was assessed using the AIMS, prior and at follow-up 3 months after discharge. Parents of all groups were contacted at 1, 4, and 8 weeks after discharge to assess daily tummy time and time spent in different positions. Forty-nine infants returned for follow-up. Results showed no difference between groups regarding reported tummy time. However, infants spending > 15 min daily on their tummy had a significantly greater improvement in AIMS scores than those spending < 15 min, and infants of both intervention groups showed a trend towards greater change in AIMS scores compared to infants of the control group.

Strengths of this study were its prospective RCT design with defined inclusion and exclusion criteria and the AIMS as a standardized motor outcome at baseline and follow-up. The trial’s analysis and results were reported in detail. Limitations are the unspecific description of the randomization process, missing details about blinding, and the reduced sample size at follow-up that limit generalization from the results.

Fourdain et al. [26] included 29 infants with CHD in their retrospective cohort study in Canada between 2013 and 2016. They aimed to relate developmental trajectories of gross motor skills to the number of physiotherapy sessions. They divided infants into three groups according to their motor performance at the age of 4 months: a no-intervention group (control group with *n* = 6) performing equal to or above the 10th percentile rank on the AIMS. The other two groups, an occasional (*n* = 13) and a regular intervention group (*n* = 10), included infants performing below the 10th percentile rank on the AIMS. The intervention started at 4 months and ended at 8 months of age. The occasional intervention group received 1–2 physiotherapy sessions. The regular intervention group received 3–6 sessions at the neurocardiac clinic and continued in an outside setting thereafter. The frequency of therapy sessions was decided by clinical judgment, AIMS scores, and the presence of atypical muscle tone. Both intervention groups received the same type of intervention, with one session lasting 60 min. These always included a parental coaching session and parental guidance in observing and correcting postural and/or functional compensations. Additionally, physiotherapy included strengthening and functional activity-based exercises with functional goals. The AIMS was assessed at baseline 4 months of age, and the BSID-III were used in follow-ups at 12 and 24 months of age. The B-III scores of neither the no-intervention nor the occasional intervention groups changed significantly between 12 and 24 months. Infants with motor delay at 4 months of age showed improvements in their gross motor scores between 12 and 24 months of age after regular physical therapy. However, their performance did not differ from that of the no-intervention group at 24 months. The possibilities cannot be excluded that this improvement was due to normal development or spontaneous recovery after surgery.

Strengths of this study were its clearly defined inclusion and exclusion criteria, its control group, standardized motor outcome, and the follow-ups at 12 and 24 months of age. Limitations were the retrospective design and the lack of information about the frequency of physical therapy provided at the regional pediatric centers. The sample size of the study limits generalization from the results.

Haseba et al. [28] evaluated the influence of early postoperative physiotherapy on gross motor outcome in patients with CHD in their retrospective cohort study without a control group. They recruited 51 infants with CHD in Japan between 2013 and 2015. Two subgroups of infants between 3 months and 3 years of age were formed: 25 with cyanotic CHD and 26 with acyanotic CHD. The intervention started in the hospital at an average of 5 days after cardiac surgery and lasted an average of 18 days. All patients showed decreased gross motor ability after surgery. Children received physiotherapy 1–3 times a day, 20–60 min each, and 5–6 days per week. The treatment consisted of respiratory exercises to prevent respiratory complications and exercises to improve gross motor abilities. A nine-grade program of exercises was conducted according to the children’s activity levels. Exercises ranged from passive or active assistive movements to exercises that promoted children’s activity in supine, prone, sitting, standing, and walking with or without support. Haseba et al. [28] developed a nine-grade mobility assessment scale to evaluate children’s motor outcome at three time points: preoperative, at physiotherapy initiation, and before discharge. At discharge, 88% of the recruited cyanotic CHD patients and 96.2% of the acyanotic CHD patients had improved their preoperative mobility grade. The cyanotic group had significantly longer recovery periods compared than the acyanotic group. These authors suggested that initiating intervention soon after surgery reduces the duration of ICU, hospital stay, and mechanical ventilation.

Strengths of this study included the relatively large number of study participants and the blinding of the assessors. Researchers used strict inclusion and exclusion criteria and reported their intervention program comprehensibly and their analysis and results in detail. Weaknesses are the lack of a control group and the use of a nine-grade assessment scale that is not a standardized or validated outcome tool. No information was provided on how to perform and rate this scale.

Long et al. [27] described the challenges of trying to implement an early intervention program for infants with CHD in Australia. They aimed to recruit CHD infants and match them with historical controls. The intervention started at 3 months of age and aimed to correct musculoskeletal impairments and gross motor delay and provide individualized developmental support to caregivers. Infants likely to attend the hospital received formalized and standardized outpatient physiotherapy. Infants not able to attend the hospital were offered a single educational physiotherapy session. This research group planned to measure outcomes at baseline and after the end of the intervention at 8 and 12 months. They managed to recruit 12 infants, of which only 6 could ultimately be included. Recruitment was stopped prematurely because several objections to participation arose from both the families and service providers. The effectiveness of intervention services could not be evaluated with the data collected. Long et al. [27] showed that family acceptance is limited when physical therapy is offered in a tertiary setting under the current model of care in Australia.

Major strengths of this study were its prospective design; the blinding of the assessors to diagnosis, history, and intervention group; and the use of standardized outcome measures such as the AIMS and the Ages and Stages Questionnaire 3rd Edition (ASQ-3) [29], a parent-completed screening questionnaire for identifying developmental delay and disorders. Researchers precisely described their difficulties in evaluating the effectiveness of the intervention in CHD infants and factors leading to nonparticipation in this cohort. Limitations of this study include a lack of details about intervention type and frequency and session duration. Thus, it is difficult to conclude what findings might be expected of similar studies conducted elsewhere.

## Discussion

Our study systematically reviewed four studies [25–28] that investigated early motor interventions in infants with CHD after open-heart surgery. As previously expected, our review confirmed that only few studies examined early motor intervention in CHD infants after open-heart surgery. Over the last 8 years (2015–2023), only four studies were published, with low to high methodological quality. Because their level of evidence is low to high, their results must be interpreted with caution.

### Methodological considerations

Only one study had high methodological quality [25], one had moderate [26], and two had low methodological quality [27, 28] often related to the study design and small sample sizes.

Uzark et al. [25], Fourdain et al. [26], and Haseba et al. [28] showed good representativeness for their cohorts due to their relatively large number of patients and well-defined inclusion and exclusion criteria. Only one study [25] randomized participants; the other studies were prone to selection bias. Fourdain et al. [26] included a control group, but this was only small in size. Two studies [25, 26] showed a high risk of performance and assessment bias as they did not blind the assessors. Blinding of families and physiotherapists is obviously not possible in these interventional studies. Long et al. [27] and Haseba et al. [28] blinded assessors to the intervention. Uzark et al. [25], Fourdain et al. [26], and Haseba et al. [28] described the statistical analyses.

### Effects of early intervention of studies

The interventions are difficult to compare. No conclusion can be drawn about the effect of early intervention in infants with CHD. This is due to the heterogeneity of studies investigating these interventions, their small group sizes, and their level of evidence. Nevertheless, certain trends can be observed. First, all the studies investigated the influence of early physical therapy in infants with CHD. The studies by Uzark et al. [25], Fourdain et al. [26], and Haseba et al. [28] focused on strengthening and functional activities, which corresponds with the current state of knowledge for early intervention therapy in high-risk infants [11].

Second, no further conclusions can be drawn about the optimal duration and frequency of therapy sessions and when best to start with intervention. The duration of a single therapy session was similar in all studies, approximately 60 min. However, the frequency varied significantly. Some treatments were performed several times or once daily, others a few times a week, and some only a few in total. All four studies started the intervention after surgery. The interventions’ duration ranged from an average of 18 days to 4 months. Accordingly, the literature provides no clear recommendations for early intervention in infants with CHD.

Third, the feasibility and implementation of the interventions were challenging. Long et al. [27] had to stop their study early because of objections from both the family and the service providers. Meyer et al. [30] observed that the major challenges in most studies are adherence and compliance. Conducting intervention studies in infants is challenging per se and may be another reason why the literature in this area is so sparse.

### Outcome measures of studies

All authors except Haseba et al. [28] used standardized motor assessments like the AIMS and the BSID-III. As studies did not assess motor outcomes at same time points, it proved difficult to compare results. All studies had a baseline measure at time of therapy initiation, but only Fourdain et al. [26] evaluated any follow-up measurements to assess post-intervention outcome. It is quite possible that intervention effects need time to become visible and thus can only be detected over time. It is therefore important to perform adequate follow-ups to identify improvements resulting from interventions. Additionally, the effects of early intervention may overlap with spontaneous biological development and the effects of environmental factors in early childhood, which make intervention effects hard to identify.

Secondary outcomes such as infants’ language and cognitive development and parental quality of life were not reported across the studies. Parental involvement and consulting are described in the following section.

### Parental involvements and consulting

Included studies did either not report outcomes of parental quality of life or only described parental involvement in a qualitative way. Uzark et al. [25] and Fourdain et al. [26] explicitly involved parents in the performance of the intervention and provided parents with home-based training. Long et al. [27] and Haseba et al. [28] also evaluated parents’ opinions about their infants’ physical therapy intervention and gross motor abilities. Haseba et al. [28] suggested that postoperative physical therapy programs might reduce parents’ postoperative anxiety and might educate parents to integrate home-based interventions into their family life. Some literature has examined the importance of family-centered intervention [31, 32]. However, this only seems to be partially implemented in children with CHD. Parents of infants with CHD often experience high levels of stress from early diagnosis on, during the hospitalization period and later. This affects parents’ quality of life and mental health, which in turn influences children’s health [33]. Research reveals that parental mental health and family functioning might have greater influence on child than the physiological impact of CHD itself [34–36]. This underlines our observation that a family-based program is one of the essential aspects of early intervention. Future studies not only should involve parents in the implementation of early intervention but also should focus on parents’ outcome and quality of life.

### Strengths and limitations of this review

To the best of our knowledge, this is the first systematic review investigating early motor intervention programs for infants with CHD after open-heart surgery that additionally describes if parents were involved in the intervention process. Nonetheless, the results of this review except of one are inconclusive due to the low to moderate level of evidence of the studies included. We only considered literature published in English or German and thus might have missed other motor intervention programs for infants with CHD, which might have influenced our results. Additionally, we restricted our systematic review to infants with a mean age below 12 months. We are aware that various factors influence and promote infants’ development. Other approaches, such as family-based psychosocial and mental health interventions, are vital to improve parental and infant’s well-being [35, 37]. However, we decided to direct our interest to motor interventions promoting infants’ motor development. All studies included in this review examined the influence of physiotherapy on motor outcomes; no studies were found that investigated other types of motor intervention.

In addition to the included studies, we extracted relevant information from protocols trials and different case studies. The RCT by Lambert et al. [38], e.g., is currently investigating influence of passive movement on growth, neurobehavior, neurodevelopment, and bone mineral density in infants with univentricular CHD. The RCT protocol by Du et al. [39] describes a structured program of passive movement and active exercise for infants after cardiac catheterization aiming to improve motor development, growth, heart function, and bone quality. The single case study by Gallagher et al. [40] even formed the basis for the study by Fourdain et al. [26] we included in our review.

## Conclusions

All studies reported improvements in CHD children’s motor development. Although the heterogeneity of study designs, interventions, and outcomes do not allow meaningful conclusion, they do provide suggestions for future research. The body of evidence on early intervention is comprehensive and supports the importance for infants at risk for developmental impairments, such as infants born preterm and those born with hypoxic-ischemic encephalopathy [10, 41, 42]. Thus, literature exists for other at-risk infants, and evidence is accumulating that such intervention programs may be applicable or adaptable to other at-risk populations, including CHD infants.

Nonetheless, it is evident that infants with CHD often show delayed motor development [14, 43, 44]. Reduced physical activity, starting early, most likely continues during childhood and later. Motor developmental delay is the first developmental problem that becomes apparent in infants with CHD [43], and it seems clear that these infants may benefit from early motor intervention. Seeking to prevent problems before they manifest and improve existing problems should reduce difficulties later in life. However, early interventions focusing on infants with CHD to promote motor development are sparse. In particular, studies with high methodological quality and that involve parents are needed to investigate early motor intervention in this at-risk population.

### Supplementary Information

**Additional file 1. **Initial search strategy.

**Additional file 2. **Update search strategy.

**Additional file 3. **Prisma 2020 abstract checklist.

**Additional file 4. **PRISMA 2020 checklist.

## Data Availability

The datasets used and analyzed in this systematic review are available from the corresponding author on reasonable request.

## Abbreviations

RK: Rahel Kaeslin
EM: Elena Mitteregger
BL: Bea Latal
ASQ3: Ages and Stages Questionnaire 3rd Edition
AIMS: Alberta Infant Motor Scale
BSID-III: Bayley Scales of Infant and Toddler Development, Third Edition
CHD: Congenital heart disease
ICU: Intensive care unit
TIMP: Test of Infant Motor Performance
