# Supplementary material for: A systematic review of early motor interventions for infants with congenital heart disease and open-heart surgery
Source: Syst Rev. 2023 Aug 25;12:149. doi: 10.1186/s13643-023-02320-3 (PMC10463862; doi:10.1186/s13643-023-02320-3)
Supplement: Supplementary file 1 — Additional file 1. Initial search strategy. [file 13643_2023_2320_MOESM1_ESM.pdf]

Suchprotokoll zur Studie Early motor interventions in children with CHD

Suchprotokolle:

CINAHL_Print Search History EBSCOhost....	12.05.2020
Exported HTML _ Embase.pdf	13.05.2020
Medline_Print Search History EBSCOhost....	12.05.2020
Pedro.pdf	13.05.2020
PsycINFO_Print Search History EBSCOhost...	12.05.2020
Scopus - Advanced search.pdf	13.05.2020
Search Manager _ Cochrane Library.pdf	13.05.2020

	**Deduplication**	
	Before	after
Medline	114	114
EMBASE	200	158
CINAHL	57	12
Cochrane	14	8
PsycINFO	10	1
PEDRO	1	0
Scopus	175	57
Pool	571	**350**

Reference files: Early motor interventions in children with CHD.enlx

Before:

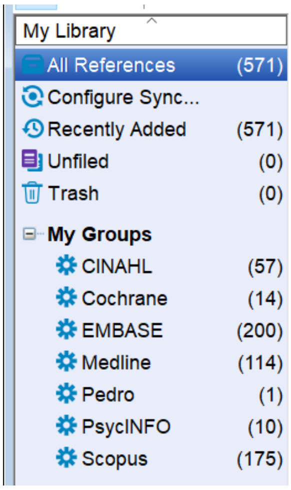

after:

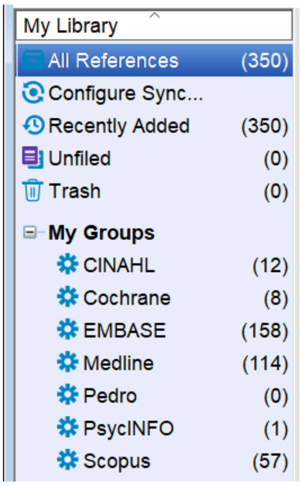

Tuesday, May 12, 2020 3:52:10 AM

#	Query	Limiters/Expanders	Last Run Via	Results
S8	S3 AND S4 AND S5 AND S6	Limiters - Language: English, French, German, Italian<br>Expanders - Apply equivalent subjects<br>Search modes - Find all my search terms	Interface - EBSCOhost Research Databases<br>Search Screen - Advanced Search<br>Database - MEDLINE	114
S7	S3 AND S4 AND S5 AND S6	Expanders - Apply equivalent subjects<br>Search modes - Find all my search terms	Interface - EBSCOhost Research Databases<br>Search Screen - Advanced Search<br>Database - MEDLINE	117
S6	(MH "Treatment Outcome+") OR (MH "Outcome and Process Assessment, Health Care+") OR (MH "Upper Extremity") OR (MH "Lower Extremity+") OR (MH "Motor Skills") OR (MH "Motor Skills Disorders") OR (MH "Gait Disorders, Neurologic+") OR TI (motor N3 (skill* OR ability* OR develop* OR outcome* OR function* OR gross OR fine OR activit* OR performance OR improve*)) OR TI ((limb OR extremit* OR hand OR foot) N3 (function OR skill* OR ability*)) OR TI (outcome OR movement OR gait) OR AB (motor N3 (skill* OR ability* OR develop* OR outcome* OR function* OR gross OR fine OR activit* OR performance OR improve*)) OR AB ((limb OR extremit* OR hand OR foot) N3 (function OR skill* OR ability*)) OR AB (outcome OR movement OR gait)	Expanders - Apply equivalent subjects<br>Search modes - Find all my search terms	Interface - EBSCOhost Research Databases<br>Search Screen - Advanced Search<br>Database - MEDLINE	Display
S5	(MH "Heart Defects,	Expanders - Apply	Interface - EBSCOhost Research	Display

	Congenital+/RH") OR (MH "Physical Therapy Modalities+") OR (MH "Physical Therapy Specialty") OR (MH "Exercise Therapy+") OR (MH "Physical Therapists") OR (MH "Occupational Therapists") OR (MH "Occupational Therapy") OR (MH "Restraint, Physical+") OR (MH "Early Intervention, Educational") OR TI ((physical OR occupational OR constraint-induced OR neurodevelopment* OR neuro-development* OR motor OR movement OR exercise) N3 (train* OR therap* OR intervention* OR treat* OR support* OR enhance*)) OR TI (physiotherap* OR NDT OR bobath* OR Vojta*) OR AB ((physical OR occupational OR constraint-induced OR neurodevelopment* OR neuro-development* OR motor OR movement OR exercise) N3 (train* OR therap* OR intervention* OR treat* OR support* OR enhance*)) OR AB (physiotherap* OR NDT OR bobath* OR Vojta*)	equivalent subjects<br>Search modes - Find all my search terms	Databases<br>Search Screen - Advanced Search<br>Database - MEDLINE	
S4	(MH "Pediatrics+") OR (MH "Infant+") OR (MH "Intensive Care Units, Pediatric+") OR TI (infant* OR baby OR babies OR neonat* OR newborn OR preterm OR prematur* OR "after birth") OR AB (infant* OR baby OR babies OR neonat* OR newborn OR preterm OR prematur* OR "after birth")	Expanders - Apply equivalent subjects<br>Search modes - Find all my search terms	Interface - EBSCOhost Research Databases<br>Search Screen - Advanced Search<br>Database - MEDLINE	Display
S3	(MH "Heart Defects, Congenital+") OR (MH	Expanders - Apply equivalent subjects	Interface - EBSCOhost Research Databases	Display

"Heart Diseases+/CN") OR TI (((congenital* OR hereditary OR inborn) AND ((heart* or cardiac* or coronary or septal* or aortopulmonary or aorticopulmonary or atrial or ventricular or intraventricular) N3 (defect* or disease* OR malformation* or abnormal* or anomal*))) OR (digeorge N1 (syndrome* or anomal* or sequenc*)) OR (transpos* N3 (arteries or artery or vessel*)) OR (alagille N2 syndrome) OR ("arteriohepatic dysplasia*" or "gonadal dysgenesis" or "subdivided left atrium*") OR ((cardiovertebral OR "pharyngeal pouch" OR "thymic aplasia" OR "conotruncal anomaly face" OR turner* OR noonan OR barth OR velo* OR kartagener* OR siewert* OR scimitar OR lutembacher* OR leopard or "multiple lentigines" OR marfan*) N3 syndrome*) OR ("hepatic hypoplasia" or "arteriohepatic dysplasia*" or "bicuspid aortic valve") OR (taussig* N2 anomal*) OR ((pulmon* or aortic or subaortic or valve or mitral) N1 stenosis) OR ((aortic or aorta*) N3 coarctation*) OR (ventricular N2 dysplasia*) OR ("cor triatriatum" or cortriatriatum or "triatrial heart*") OR ("myocardial bridging*" or "crisscross heart*" or "criss-cross heart*") OR (dextrocardia* or "kartagener* triad" or "primary ciliary dyskinesia") OR ("patent ductus	Search modes - Find all my search terms	Search Screen - Advanced Search<br>Database - MEDLINE

arteriosus" or "anomalous pulmonary venous connection" or "double inlet left ventricle" or "double outlet right ventricle" or "interrupted aortic arch") OR ("ebstein* anomaly" or "ebstein* malformation*" or "ectopia cordis") OR (eisenmenger* N1 (complex or syndrome)) OR ("persistent truncus arteriosus" or "persistent ostium primum") OR ("endocardial cushion defect*" or "atrioventricular canal") OR ("foramen oval*") OR (heart N3 hypoplas*) OR ((noncompaction OR "non compaction") N3 "ventricular myocardium") OR (levocardia) OR (((tetralogy or trilogy or syndrome) N2 fallot*) or cantrell* or shon?s) OR ((tricuspid OR valve OR pulmonary) N1 atresia*) or ("absent right atrioventricular connection" OR "single ventricle physiology" or GUCH or "cavopulmonary connection") OR ((bonnevie N2 (syndrome* or status)) or "polynesian bronchiectas*")) OR AB (((congenital* OR hereditary OR inborn) AND ((heart* or cardiac* or coronary or septal* or aortopulmonary or aorticopulmonary or atrial or ventricular or intraventricular) N3 (defect* or disease* OR malformation* or abnormal* or anomal*))) OR (digeorge N1 (syndrome* or anomal* or sequenc*)) OR (transpos* N3 (arteries or artery or vessel*)) OR (alagille N2

syndrome) OR
("arteriohepatic dysplasia*"
or "gonadal dysgenesis" or
"subdivided left atrium*") OR
((cardiovertebral OR
"pharyngeal pouch" OR
"thymic aplasia" OR
"conotruncal anomaly face"
OR turner* OR noonan OR
barth OR velo* OR
kartagener* OR siewert* OR
scimitar OR lutembacher*
OR leopard or "multiple
lentigines" OR marfan*) N3
syndrome*) OR ("hepatic
hypoplasia" or
"arteriohepatic dysplasia*" or
"bicuspid aortic valve") OR
(taussig* N2 anomal*) OR
((pulmon* or aortic or
subaortic or valve or mitral)
N1 stenosis) OR ((aortic or
aorta*) N3 coarctation*) OR
(ventricular N2 dysplasia*)
OR ("cor triatriatum" or
cortriatriatum or "triatrial
heart*") OR ("myocardial
bridging*" or "crisscross
heart*" or "criss-cross
heart*") OR (dextrocardia* or
"kartagener* triad" or
"primary ciliary dyskinesia")
OR ("patent ductus
arteriosus" or "anomalous
pulmonary venous
connection" or "double inlet
left ventricle" or "double
outlet right ventricle" or
"interrupted aortic arch") OR
("ebstein* anomaly" or
"ebstein* malformation*" or
"ectopia cordis") OR
(eisenmenger* N1 (complex
or syndrome)) OR
("persistent truncus
arteriosus" or "persistent
ostium primum") OR
("endocardial cushion
defect*" or "atrioventricular

	canal") OR ("foramen oval*") OR (heart N3 hypoplas*) OR ((noncompaction OR "non compaction") N3 "ventricular myocardium") OR (levocardia) OR (((tetralogy or trilogy or syndrome) N2 fallot*) or cantrell* or shon?s) OR ((tricuspid OR valve OR pulmonary) N1 atresia*) or ("absent right atrioventricular connection" OR "single ventricle physiology" or GUCH or "cavopulmonary connection") OR ((bonnevie N2 (syndrome* or status)) or "polynesian bronchiectas*"))			
S2	(MH "Infant+")	Expanders - Apply equivalent subjects<br>Search modes - Find all my search terms	Interface - EBSCOhost Research Databases<br>Search Screen - Advanced Search<br>Database - MEDLINE	1,129,818
S1	(MH "Outcome and Process Assessment, Health Care+")	Expanders - Apply equivalent subjects<br>Search modes - Find all my search terms	Interface - EBSCOhost Research Databases<br>Search Screen - Advanced Search<br>Database - MEDLINE	1,143,274

Embase®

Embase Session Results (13 May 2020)

No.	Query	Results
#9	#7 OR #8	200
#8	#1 AND #2 AND #3 AND #4 NOT [conference abstract]/lim AND ([english]/lim OR [french]/lim OR [german]/lim OR [italian]/lim)	174
#7	#1 AND #2 AND #3 AND #4 AND [conference abstract]/lim AND [2018-2020]/py AND ([english]/lim OR [french]/lim OR [german]/lim OR [italian]/lim)	26
#6	#1 AND #2 AND #3 AND #4 AND ([english]/lim OR [french]/lim OR [german]/lim OR [italian]/lim)	251
#5	#1 AND #2 AND #3 AND #4	252
#4	'treatment outcome'/exp OR 'outcome assessment'/exp OR 'upper limb'/exp OR 'lower limb'/exp OR 'psychomotor disorder'/exp OR 'neurologic gait disorder'/exp OR ((motor NEAR/3 (skill* OR ability* OR develop* OR outcome* OR function* OR gross OR fine OR activit* OR performance OR improve*)):ti,ab) OR (((limb OR extremit* OR hand OR foot) NEAR/3 (function OR skill* OR ability*)):ti,ab) OR outcome:ti,ab OR movement:ti,ab OR gait:ti,ab	3614317
#3	'congenital heart disease'/exp/dm_rh OR 'physiotherapy'/exp OR 'kinesiotherapy'/exp OR 'exercise therapy in infancy and childhood'/exp OR 'exercise'/exp OR 'physiotherapist'/exp OR 'occupational therapy'/exp OR 'occupational therapist'/exp OR 'early childhood intervention'/exp OR (((physical OR occupational OR 'constraint induced' OR neurodevelopment* OR 'neuro development*' OR motor OR movement OR exercise) NEAR/3 (train* OR therap* OR intervention* OR treat* OR support* OR enhance*)):ti,ab) OR physiotherap*:ti,ab OR ndt:ti,ab OR bobath*:ti,ab OR vojta*:ti,ab	572502
#2	'pediatrics'/exp OR 'infant'/exp OR infant*:ti,ab OR baby:ti,ab OR babies:ti,ab OR neonat*:ti,ab OR newborn:ti,ab OR preterm:ti,ab OR prematur*:ti,ab OR 'after birth':ti,ab	1714184
#1	'congenital heart disease'/exp OR 'heart disease'/exp/dm_cn OR ((congenital*:ti,ab OR hereditary:ti,ab OR inborn:ti,ab) AND (((heart* OR cardiac* OR coronary OR septal* OR aortopulmonary OR aorticopulmonary OR atrial OR ventricular OR intraventricular) NEAR/3 (defect* OR disease* OR malformation* OR abnormal* OR anomal*)):ti,ab)) OR ((digeorge NEAR/1 (syndrome* OR anomal* OR sequenc*)):ti,ab) OR ((transpos* NEAR/3 (arteries OR artery OR vessel*)):ti,ab) OR ((alagille NEAR/2 syndrome):ti,ab) OR 'gonadal dysgenesis':ti,ab OR 'subdivided left atrium*':ti,ab OR (((cardiovertebral OR 'pharyngeal pouch' OR 'thymic aplasia' OR 'conotruncal anomaly face' OR turner* OR noonan OR barth OR velo* OR kartagener* OR siewert* OR scimitar OR lutembacher* OR leopard OR 'multiple lentigines' OR marfan*) NEAR/3 syndrome*):ti,ab) OR 'hepatic hypoplasia':ti,ab OR 'arteriohepatic dysplasia*':ti,ab OR 'bicuspid aortic valve':ti,ab OR ((taussig* NEAR/2 anomal*):ti,ab) OR (((pulmon* OR aortic OR subaortic OR valve OR mitral) NEAR/1 stenosis):ti,ab) OR (((aortic OR aorta*) NEAR/3 coarctation*):ti,ab) OR ((ventricular NEAR/2 dysplasia*):ti,ab) OR 'cor triatriatum':ti,ab OR cortriatriatum:ti,ab OR 'triatrial heart*':ti,ab OR 'myocardial bridging*':ti,ab OR 'crisscross heart*':ti,ab OR 'criss-cross heart*':ti,ab OR dextrocardia*:ti,ab OR 'kartagener* triad':ti,ab OR 'primary ciliary dyskinesia':ti,ab OR 'patent ductus arteriosus':ti,ab OR 'anomalous pulmonary venous connection':ti,ab OR 'double inlet left ventricle':ti,ab OR 'double outlet right ventricle':ti,ab OR 'interrupted aortic arch':ti,ab OR 'ebstein* anomaly':ti,ab OR 'ebstein* malformation*':ti,ab OR 'ectopia cordis':ti,ab OR ((eisenmenger* NEAR/1 (complex OR syndrome)):ti,ab) OR 'persistent truncus arteriosus':ti,ab OR 'persistent ostium primum':ti,ab OR 'endocardial cushion defect*':ti,ab OR 'atrioventricular canal':ti,ab OR 'foramen oval*':ti,ab OR ((heart NEAR/3 hypoplas*):ti,ab) OR (((noncompaction OR 'non compaction') NEAR/3 'ventricular myocardium'):ti,ab) OR levocardia:ti,ab OR (((tetralogy OR trilogy OR syndrome) NEAR/2 fallot*):ti,ab) OR cantrell*:ti,ab OR shon?s:ti,ab OR (((tricuspid OR valve OR pulmonary) NEAR/1 atresia*):ti,ab) OR 'absent right atrioventricular connection':ti,ab OR 'single ventricle physiology':ti,ab OR guch:ti,ab OR 'cavopulmonary connection':ti,ab OR ((bonnevie NEAR/2 (syndrome* OR status)):ti,ab) OR 'polynesian bronchiectas*':ti,ab	286560

ELSEVIER

© 2020 Elsevier Life Sciences IP Limited except certain content provided by third parties.
Embase is a trade mark of Elsevier Life Sciences IP Limited. RELX Group and the RE symbol are trade marks of RELX Group plc, used under license.

Cookies
Our site uses cookies to improve your experience. You can find out more about our use of cookies in About Cookies, including instructions on how to turn off cookies if you wish to do so. By continuing to browse this site you agree to us using cookies as described in About Cookies.

I accept

Cochrane Library

Explore new Cochrane Library features **here**.

We noticed your browser language is German.
You can select your preferred language at the top of any page, and you will see translated Cochrane Review sections in this language. Change to **German.**

Advanced Search

Search manager

Save this search | View saved searches | ? Search help

Print

#	Search		Results
#1	((congenital* OR hereditary OR inborn):ti,ab,kw AND ((heart* or cardiac* or coronary or septal* or aortopulmonary or aorticopulmonary or atrial or ventricular or intraventricular) NEAR/3 (defect* or disease* OR malformation* or abnormal* or anomal*)):ti,ab,kw) OR (digeorge NEAR/1 (syndrome* or anomal* or sequenc*)):ti,ab,kw OR (transpos* NEAR/3 (arteries or artery or vessel*)):ti,ab,kw OR (alagille NEAR/2 syndrome):ti,ab,kw OR ("arteriohepatic dysplasia*" or "gonadal dysgenesis" or "subdivided left atrium*"):ti,ab,kw OR ((cardiovertebral OR "pharyngeal pouch" OR "thymic aplasia" OR "conotruncal anomaly face" OR turner* OR noonan OR barth OR velo* OR kartagener* OR siewert* OR scimitar OR lutembacher* OR leopard or "multiple lentigines" OR marfan*) NEAR/3 syndrome*):ti,ab,kw OR ("hepatic hypoplasia" or "arteriohepatic dysplasia*" or "bicuspid aortic valve"):ti,ab,kw OR (taussig* NEAR/2 anomal*):ti,ab,kw OR ((pulmon* or aortic or subaortic or valve or mitral) NEAR/1 stenosis):ti,ab,kw OR ((aortic or aorta*) NEAR/3 coarctation*):ti,ab,kw OR (ventricular NEAR/2 dysplasia*):ti,ab,kw OR ("cor triatriatum" or cortriatriatum or "triatrial heart*"):ti,ab,kw OR ("myocardial bridging*" or "crisscross heart*" or "criss-cross heart*"):ti,ab,kw OR (dextrocardia* or "kartagener* triad" or "primary ciliary dyskinesia"):ti,ab,kw OR ("patent ductus arteriosus" or "anomalous pulmonary venous connection" or "double inlet left ventricle" or "double outlet right ventricle" or "interrupted aortic arch"):ti,ab,kw OR ("ebstein* anomaly" or "ebstein* malformation*" or "ectopia cordis"):ti,ab,kw OR (eisenmenger* NEAR/1 (complex or syndrome)):ti,ab,kw OR ("persistent truncus arteriosus" or "persistent ostium primum"):ti,ab,kw OR ("endocardial cushion defect*" or "atrioventricular canal"):ti,ab,kw OR ("foramen oval*"):ti,ab,kw OR (heart NEAR/3 hypoplas*):ti,ab,kw OR ((noncompaction OR "non compaction") NEAR/3 "ventricular myocardium"):ti,ab,kw OR (levocardia):ti,ab,kw OR (((tetralogy or trilogy or syndrome) NEAR/2 fallot*) or cantrell* or shon?s):ti,ab,kw OR ((tricuspid OR valve OR pulmonary) NEAR/1 atresia*):ti,ab,kw or ("absent right atrioventricular connection" OR "single ventricle physiology" or GUCH or "cavopulmonary connection"):ti,ab,kw OR ((bonnevie NEAR/2 (syndrome* or status)) or "polynesian bronchiectas*"):ti,ab,kw	Limits	5742
#2	(infant* OR baby OR babies OR neonat* OR newborn OR preterm OR prematur* OR "after birth"):ti,ab,kw	Limits	82809
#3	((physical OR occupational OR constraint-induced OR neurodevelopment* OR neuro-development* OR motor OR movement OR exercise) NEAR/3 (train* OR therap* OR intervention* OR treat* OR support* OR enhance*)):ti,ab,kw OR (physiotherap* OR NDT OR bobath* OR Vojta*):ti,ab,kw	Limits	63295
#4	(motor NEAR/3 (skill* OR ability* OR develop* OR outcome* OR function* OR gross OR fine OR activit* OR performance OR improve*)):ti,ab,kw OR ((limb OR extremit* OR hand OR foot) NEAR/3 (function OR skill* OR ability*)):ti,ab,kw OR (outcome OR movement OR gait):ti,ab,kw	Limits	495530
#5	#1 AND #2 AND #3 AND #4	Limits	14

Tuesday, May 12, 2020 3:56:20 AM

#	Query	Limiters/Expanders	Last Run Via	Results
S6	S1 AND S2 AND S3 AND S4	Limiters - Language: English, French, German, Italian<br>Expanders - Apply equivalent subjects<br>Search modes - Find all my search terms	Interface - EBSCOhost Research Databases<br>Search Screen - Advanced Search<br>Database - CINAHL with Full Text	57
S5	S1 AND S2 AND S3 AND S4	Expanders - Apply equivalent subjects<br>Search modes - Find all my search terms	Interface - EBSCOhost Research Databases<br>Search Screen - Advanced Search<br>Database - CINAHL with Full Text	59
S4	(MH "Treatment Outcomes+") OR (MH "Outcome Assessment") OR (MH "Upper Extremity+") OR (MH "Lower Extremity+") OR (MH "Motor Skills+") OR (MH "Motor Skills Disorders") OR (MH "Gait Disorders, Neurologic+") OR TI (motor N3 (skill* OR ability* OR develop* OR outcome* OR function* OR gross OR fine OR activit* OR performance OR improve*)) OR TI ((limb OR extremit* OR hand OR foot) N3 (function OR skill* OR ability*)) OR TI (outcome OR movement OR gait) OR AB (motor N3 (skill* OR ability* OR develop* OR outcome* OR function* OR gross OR fine OR activit* OR performance OR improve*)) OR AB ((limb OR extremit* OR hand OR foot) N3 (function OR skill* OR ability*)) OR AB (outcome OR movement OR gait)	Expanders - Apply equivalent subjects<br>Search modes - Find all my search terms	Interface - EBSCOhost Research Databases<br>Search Screen - Advanced Search<br>Database - CINAHL with Full Text	1,045,325
S3	(MH "Heart Defects,	Expanders - Apply	Interface - EBSCOhost Research	260,290

	Congenital+/RH") OR (MH "Physical Therapy+") OR (MH "Pediatric Physical Therapy") OR (MH "Physical Therapists") OR (MH "Therapeutic Exercise+") OR (MH "Restraint, Physical") OR (MH "Early Childhood Intervention") OR (MH "Occupational Therapy") OR (MH "Pediatric Occupational Therapy") OR (MH "Occupational Therapists") OR TI ((physical OR occupational OR constraint-induced OR neurodevelopment* OR neuro-development* OR motor OR movement OR exercise) N3 (train* OR therap* OR intervention* OR treat* OR support* OR enhance*)) OR TI (physiotherap* OR NDT OR bobath* OR Vojta*) OR AB ((physical OR occupational OR constraint-induced OR neurodevelopment* OR neuro-development* OR motor OR movement OR exercise) N3 (train* OR therap* OR intervention* OR treat* OR support* OR enhance*)) OR AB (physiotherap* OR NDT OR bobath* OR Vojta*)	equivalent subjects<br>Search modes - Find all my search terms	Databases<br>Search Screen - Advanced Search<br>Database - CINAHL with Full Text	
S2	(MH "Pediatrics+") OR (MH "Infant+") OR (MH "Intensive Care Units, Pediatric+") OR TI (infant* OR baby OR babies OR neonat* OR newborn OR preterm OR prematur* OR "after birth") OR AB (infant* OR baby OR babies OR neonat* OR newborn OR preterm OR prematur* OR "after birth")	Expanders - Apply equivalent subjects<br>Search modes - Find all my search terms	Interface - EBSCOhost Research Databases<br>Search Screen - Advanced Search<br>Database - CINAHL with Full Text	388,128
S1	(MH "Heart Defects,	Expanders - Apply	Interface - EBSCOhost Research	41,567

Congenital+") OR TI (((congenital* OR hereditary OR inborn) AND ((heart* or cardiac* or coronary or septal* or aortopulmonary or aorticopulmonary or atrial or ventricular or intraventricular) N3 (defect* or disease* OR malformation* or abnormal* or anomal*))) OR (digeorge N1 (syndrome* or anomal* or sequenc*)) OR (transpos* N3 (arteries or artery or vessel*)) OR (alagille N2 syndrome) OR ("arteriohepatic dysplasia*" or "gonadal dysgenesis" or "subdivided left atrium*") OR ((cardiovertebral OR "pharyngeal pouch" OR "thymic aplasia" OR "conotruncal anomaly face" OR turner* OR noonan OR barth OR velo* OR kartagener* OR siewert* OR scimitar OR lutembacher* OR leopard or "multiple lentigines" OR marfan*) N3 syndrome*) OR ("hepatic hypoplasia" or "arteriohepatic dysplasia*" or "bicuspid aortic valve") OR (taussig* N2 anomal*) OR ((pulmon* or aortic or subaortic or valve or mitral) N1 stenosis) OR ((aortic or aorta*) N3 coarctation*) OR (ventricular N2 dysplasia*) OR ("cor triatriatum" or cortriatriatum or "triatrial heart*") OR ("myocardial bridging*" or "crisscross heart*" or "criss-cross heart*") OR (dextrocardia* or "kartagener* triad" or "primary ciliary dyskinesia") OR ("patent ductus arteriosus" or "anomalous	equivalent subjects<br>Search modes - Find all my search terms	Databases<br>Search Screen - Advanced Search<br>Database - CINAHL with Full Text

pulmonary venous connection" or "double inlet left ventricle" or "double outlet right ventricle" or "interrupted aortic arch") OR ("ebstein* anomaly" or "ebstein* malformation*" or "ectopia cordis") OR (eisenmenger* N1 (complex or syndrome)) OR ("persistent truncus arteriosus" or "persistent ostium primum") OR ("endocardial cushion defect*" or "atrioventricular canal") OR ("foramen oval*") OR (heart N3 hypoplas*) OR ((noncompaction OR "non compaction") N3 "ventricular myocardium") OR (levocardia) OR (((tetralogy or trilogy or syndrome) N2 fallot*) or cantrell* or shon?s) OR ((tricuspid OR valve OR pulmonary) N1 atresia*) or ("absent right atrioventricular connection" OR "single ventricle physiology" or GUCH or "cavopulmonary connection") OR ((bonnevie N2 (syndrome* or status)) or "polynesian bronchiectas*")) OR AB (((congenital* OR hereditary OR inborn) AND ((heart* or cardiac* or coronary or septal* or aortopulmonary or aorticopulmonary or atrial or ventricular or intraventricular) N3 (defect* or disease* OR malformation* or abnormal* or anomal*))) OR (digeorge N1 (syndrome* or anomal* or sequenc*)) OR (transpos* N3 (arteries or artery or vessel*)) OR (alagille N2 syndrome) OR

Brought to you by UZH Hauptbibliothek / Zentralbibliothek Zürich

Advanced search

Compare sources

Documents Authors Affiliations Advanced

Search tips

Enter query string

TITLE-ABS-KEY(motor W/3 (skill* OR ability* OR develop* OR outcome* OR function* OR gross OR fine OR activit* OR performance OR improve*)) OR TITLE-ABS-KEY((limb OR extremit* OR hand OR foot) W/3 (function OR skill* OR ability*)) OR TITLE-ABS-KEY(outcome OR movement OR gait)

Outline query Add Author name / Affiliation Clear form

ALL("Cognitive architectures") AND AUTHOR-NAME(smith)
TITLE-ABS-KEY(*somatic complaint wom?n) AND PUBYEAR AFT 1993
SRCTITLE(*field ornith*) AND VOLUME(75) AND ISSUE(1) AND PAGES(53-66)

Operators

Field codes

Search history

6	(TITLE-ABS-KEY (((congenital* OR hereditary OR inborn) AND ((heart* OR cardiac* OR coronary OR septal* OR aortopulmonary OR aorticopulmonary OR atrial OR ventricular OR intraventricular) W/3 (defect* OR disease* OR malformation* OR abnormal* OR anomal*))) OR (digeorge W/1 (syndrome* OR anomal* OR sequenc*)) OR (transpos* W/3 (arteries OR artery OR vessel*)) OR (alagille W/2 syndrome) OR ("arteriohepatic dysplasia*" OR "gonadal dysgenesis" OR "subdivided left atrium*") OR ((cardiovertebral OR "pharyngeal pouch" OR "thymic aplasia" OR "conotruncal anomaly face" OR turner* OR noonan OR barth OR velo* OR kartagener* OR siewert* OR scimitar OR lutembacher* OR leopard OR "multiple lentigines" OR marfan*) W/3 syndrome*) OR ("hepatic hypoplasia" OR "arteriohepatic dysplasia*" OR "bicuspid aortic valve") OR (taussig* W/2 anomal*) OR ((pulmon* OR aortic OR subaortic OR valve OR mitral) W/1 stenosis) OR ((aortic OR aorta*) W/3 coarctation*) OR (ventricular W/2 dysplasia*) OR ("cor triatriatum" OR cortriatriatum OR "triatrial heart*") OR ("myocardial bridging*" OR "crisscross heart*" OR "criss-cross heart*") OR (dextrocardia* OR "kartagener* triad" OR "primary ciliary dyskinesia") OR ("patent ductus arteriosus" OR "anomalous pulmonary venous connection" OR "double inlet left ventricle" OR "double outlet right ventricle" OR "interrupted aortic arch") OR ("ebstein* anomaly" OR "ebstein* malformation*" OR "ectopia cordis") OR (eisenmenger* W/1 (complex OR syndrome)) OR ("persistent truncus arteriosus" OR "persistent ostium primum") OR ("endocardial cushion defect*" OR "atrioventricular canal") OR ("foramen oval*") OR (heart W/3 hypoplas*) OR ((noncompaction OR "non compaction") W/3 "ventricular myocardium") OR (levocardia) OR (((tetralogy OR trilogy OR syndrome) W/2 fallot*) OR cantrell* OR shon?s) OR ((tricuspid OR valve OR pulmonary) W/1 atresia*) OR ("absent right atrioventricular connection" OR "single ventricle physiology" OR guch OR "cavopulmonary connection") OR ((bonnevie W/2 (syndrome* OR status)) OR "polynesian bronchiectas*"))) AND (TITLE-ABS-KEY (infant* OR baby OR babies OR neonat* OR newborn OR preterm OR prematur* OR "after birth")) AND (TITLE-ABS-KEY ((physical OR occupational	175 document results	

OR constraint-induced OR neurodevelopment* OR neuro-development* OR motor OR movement OR exercise) W/3 (train* OR therap* OR intervention* OR treat* OR support* OR enhance*)) OR TITLE-ABS-KEY (physiotherap* OR ndt OR bobath* OR vojta*)) AND (TITLE-ABS-KEY (motor W/3 (skill* OR ability* OR develop* OR outcome* OR function* OR gross OR fine OR activit* OR performance OR improve*)) OR TITLE-ABS-KEY ((limb OR extremit* OR hand OR foot) W/3 (function OR skill* OR ability*)) OR TITLE-ABS-KEY (outcome OR movement OR gait)) AND (LIMIT-TO (LANGUAGE , "English") OR LIMIT-TO (LANGUAGE , "German") OR LIMIT-TO (LANGUAGE , "French"))

5 (TITLE-ABS-KEY (((congenital* OR hereditary OR inborn) AND ((heart* OR cardiac* OR coronary OR septal* OR aortopulmonary OR aorticopulmonary OR atrial OR ventricular OR intraventricular) W/3 (defect* OR disease* OR malformation* OR abnormal* OR anomal*))) OR (digeorge W/1 (syndrome* OR anomal* OR sequenc*)) OR (transpos* W/3 (arteries OR artery OR vessel*)) OR (alagille W/2 syndrome) OR ("arteriohepatic dysplasia*" OR "gonadal dysgenesis" OR "subdivided left atrium*") OR ((cardiovertebral OR "pharyngeal pouch" OR "thymic aplasia" OR "conotruncal anomaly face" OR turner* OR noonan OR barth OR velo* OR kartagener* OR siewert* OR scimitar OR lutembacher* OR leopard OR "multiple lentigines" OR marfan*) W/3 syndrome*) OR ("hepatic hypoplasia" OR "arteriohepatic dysplasia*" OR "bicuspid aortic valve") OR (taussig* W/2 anomal*) OR ((pulmon* OR aortic OR subaortic OR valve OR mitral) W/1 stenosis) OR ((aortic OR aorta*) W/3 coarctation*) OR (ventricular W/2 dysplasia*) OR ("cor triatriatum" OR cortriatriatum OR "triatrial heart*") OR ("myocardial bridging*" OR "crisscross heart*" OR "criss-cross heart*") OR (dextrocardia* OR "kartagener* triad" OR "primary ciliary dyskinesia") OR ("patent ductus arteriosus" OR "anomalous pulmonary venous connection" OR "double inlet left ventricle" OR "double outlet right ventricle" OR "interrupted aortic arch") OR ("ebstein* anomaly" OR "ebstein* malformation*" OR "ectopia cordis") OR (eisenmenger* W/1 (complex OR syndrome)) OR ("persistent truncus arteriosus" OR "persistent ostium primum") OR ("endocardial cushion defect*" OR "atrioventricular canal") OR ("foramen oval*") OR (heart W/3 hypoplas*) OR ((noncompaction OR "non compaction") W/3 "ventricular myocardium") OR (levocardia) OR (((tetralogy OR trilogy OR syndrome) W/2 fallot*) OR cantrell* OR shon?s) OR ((tricuspid OR valve OR pulmonary) W/1 atresia*) OR ("absent right atrioventricular connection" OR "single ventricle physiology" OR guch OR "cavopulmonary connection") OR ((bonnevie W/2 (syndrome* OR status)) OR "polynesian bronchiectas*"))) AND (TITLE-ABS-KEY (infant* OR baby OR babies OR neonat* OR newborn OR preterm OR prematur* OR "after birth")) AND (TITLE-ABS-KEY ((physical OR occupational OR constraint-induced OR neurodevelopment* OR neuro-development* OR motor

176 document results

	OR movement OR exercise) W/3 (train* OR therap* OR intervention* OR treat* OR support* OR enhance*)) OR TITLE-ABS-KEY (physiotherap* OR ndt OR bobath* OR vojta*)) AND (TITLE-ABS-KEY (motor W/3 (skill* OR ability* OR develop* OR outcome* OR function* OR gross OR fine OR activit* OR performance OR improve*)) OR TITLE-ABS-KEY ((limb OR extremit* OR hand OR foot) W/3 (function OR skill* OR ability*)) OR TITLE-ABS-KEY (outcome OR movement OR gait))		
4	TITLE-ABS-KEY (motor W/3 (skill* OR ability* OR develop* OR outcome* OR function* OR gross OR fine OR activit* OR performance OR improve*)) OR TITLE-ABS-KEY ((limb OR extremit* OR hand OR foot) W/3 (function OR skill* OR ability*)) OR TITLE-ABS-KEY (outcome OR movement OR gait)	4,746,547 document results	
3	TITLE-ABS-KEY ((physical OR occupational OR constraint-induced OR neurodevelopment* OR neuro-development* OR motor OR movement OR exercise) W/3 (train* OR therap* OR intervention* OR treat* OR support* OR enhance*)) OR TITLE-ABS-KEY (physiotherap* OR ndt OR bobath* OR vojta*)	366,092 document results	
2	TITLE-ABS-KEY (infant* OR baby OR babies OR neonat* OR newborn OR preterm OR prematur* OR "after birth")	1,927,550 document results	
1	TITLE-ABS-KEY (((congenital* OR hereditary OR inborn) AND ((heart* OR cardiac* OR coronary OR septal* OR aortopulmonary OR aorticopulmonary OR atrial OR ventricular OR intraventricular) W/3 (defect* OR disease* OR malformation* OR abnormal* OR anomal*))) OR (digeorge W/1 (syndrome* OR anomal* OR sequenc*)) OR (transpos* W/3 (arteries OR artery OR vessel*)) OR (alagille W/2 syndrome) OR ("arteriohepatic dysplasia*" OR "gonadal dysgenesis" OR "subdivided left atrium*") OR ((cardiovertebral OR "pharyngeal pouch" OR "thymic aplasia" OR "conotruncal anomaly face" OR turner* OR noonan OR barth OR velo* OR kartagener* OR siewert* OR scimitar OR lutembacher* OR leopard OR "multiple lentigines" OR marfan*) W/3 syndrome*) OR ("hepatic hypoplasia" OR "arteriohepatic dysplasia*" OR "bicuspid aortic valve") OR (taussig* W/2 anomal*) OR ((pulmon* OR aortic OR subaortic OR valve OR mitral) W/1 stenosis) OR ((aortic OR aorta*) W/3 coarctation*) OR (ventricular W/2 dysplasia*) OR ("cor triatriatum" OR cortriatriatum OR "triatrial heart*") OR ("myocardial bridging*" OR "crisscross heart*" OR "criss-cross heart*") OR (dextrocardia* OR "kartagener* triad" OR "primary ciliary dyskinesia") OR ("patent ductus arteriosus" OR "anomalous pulmonary venous	269,533 document results	

connection" OR "double inlet left ventricle" OR "double outlet right ventricle" OR "interrupted aortic arch") OR ("ebstein* anomaly" OR "ebstein* malformation*" OR "ectopia cordis") OR (eisenmenger* W/1 (complex OR syndrome)) OR ("persistent truncus arteriosus" OR "persistent ostium primum") OR ("endocardial cushion defect*" OR "atrioventricular canal") OR ("foramen oval*") OR (heart W/3 hypoplas*) OR ((noncompaction OR "non compaction") W/3 "ventricular myocardium") OR (levocardia) OR (((tetralogy OR trilogy OR syndrome) W/2 fallot*) OR cantrell* OR shon?s) OR ((tricuspid OR valve OR pulmonary) W/1 atresia*) OR ("absent right atrioventricular connection" OR "single ventricle physiology" OR guch OR "cavopulmonary connection") OR ((bonnevie W/2 (syndrome* OR status)) OR "polynesian bronchiectas*"))

Showing all recent searches | View 5 most recent only

Top of page

Help improve Scopus

About Scopus

What is Scopus
Content coverage
Scopus blog
Scopus API
Privacy matters

Language

日本語に切り替える
切换到简体中文
切換到繁體中文
Русский язык

Customer Service

Help
Contact us

Terms and conditions Privacy policy

Copyright © Elsevier B.V. All rights reserved. Scopus® is a registered trademark of Elsevier B.V.

We use cookies to help provide and enhance our service and tailor content. By continuing, you agree to the use of cookies.

Tuesday, May 12, 2020 5:09:01 AM

#	Query	Limiters/Expanders	Last Run Via	Results
S6	S1 AND S2 AND S3 AND S4	Limiters - Language: English, French, German, Italian<br>Expanders - Apply equivalent subjects<br>Search modes - Find all my search terms	Interface - EBSCOhost Research Databases<br>Search Screen - Advanced Search<br>Database - APA PsycInfo	10
S5	S1 AND S2 AND S3 AND S4	Expanders - Apply equivalent subjects<br>Search modes - Find all my search terms	Interface - EBSCOhost Research Databases<br>Search Screen - Advanced Search<br>Database - APA PsycInfo	10
S4	DE "Treatment Outcomes" OR DE "Motor Skills" OR DE "Movement Disorders" OR TI (motor N3 (skill* OR ability* OR develop* OR outcome* OR function* OR gross OR fine OR activit* OR performance OR improve*)) OR TI ((limb OR extremit* OR hand OR foot) N3 (function OR skill* OR ability*)) OR TI (outcome OR movement OR gait) OR AB (motor N3 (skill* OR ability* OR develop* OR outcome* OR function* OR gross OR fine OR activit* OR performance OR improve*)) OR AB ((limb OR extremit* OR hand OR foot) N3 (function OR skill* OR ability*)) OR AB (outcome OR movement OR gait)	Expanders - Apply equivalent subjects<br>Search modes - Find all my search terms	Interface - EBSCOhost Research Databases<br>Search Screen - Advanced Search<br>Database - APA PsycInfo	567,824
S3	DE "Physical Treatment Methods" OR DE "Physical Restraint" OR DE "Massage" OR DE "Physical Therapy" OR DE "Exercise" OR DE "Physical Activity" OR DE "Movement Therapy" OR DE	Expanders - Apply equivalent subjects<br>Search modes - Find all my search terms	Interface - EBSCOhost Research Databases<br>Search Screen - Advanced Search<br>Database - APA PsycInfo	90,853

	"Occupational Therapy" OR TI ((physical OR occupational OR constraint-induced OR neurodevelopment* OR neuro-development* OR motor OR movement OR exercise) N3 (train* OR therap* OR intervention* OR treat* OR support* OR enhance*)) OR TI (physiotherap* OR NDT OR bobath* OR Vojta*) OR AB ((physical OR occupational OR constraint-induced OR neurodevelopment* OR neuro-development* OR motor OR movement OR exercise) N3 (train* OR therap* OR intervention* OR treat* OR support* OR enhance*)) OR AB (physiotherap* OR NDT OR bobath* OR Vojta*)			
S2	(DE "Pediatrics") OR (DE "Infant Development") OR TI (infant* OR baby OR babies OR neonat* OR newborn OR preterm OR prematur* OR "after birth") OR AB (infant* OR baby OR babies OR neonat* OR newborn OR preterm OR prematur* OR "after birth")	Expanders - Apply equivalent subjects<br>Search modes - Find all my search terms	Interface - EBSCOhost Research Databases<br>Search Screen - Advanced Search<br>Database - APA PsycInfo	149,566
S1	((DE "Congenital Disorders") AND (DE "Heart Disorders" OR DE "Angina Pectoris" OR DE "Arrhythmias (Heart)" OR DE "Coronary Thromboses" OR DE "Myocardial Infarctions")) OR TI (((congenital* OR hereditary OR inborn) AND ((heart* or cardiac* or coronary or septal* or aortopulmonary or aorticopulmonary or atrial or ventricular or	Expanders - Apply equivalent subjects<br>Search modes - Find all my search terms	Interface - EBSCOhost Research Databases<br>Search Screen - Advanced Search<br>Database - APA PsycInfo	2,450

intraventricular) N3 (defect* or disease* OR malformation* or abnormal* or anomal*))) OR (digeorge N1 (syndrome* or anomal* or sequenc*)) OR (transpos* N3 (arteries or artery or vessel*)) OR (alagille N2 syndrome) OR ("arteriohepatic dysplasia*" or "gonadal dysgenesis" or "subdivided left atrium*") OR ((cardiovertebral OR "pharyngeal pouch" OR "thymic aplasia" OR "conotruncal anomaly face" OR turner* OR noonan OR barth OR velo* OR kartagener* OR siewert* OR scimitar OR lutembacher* OR leopard or "multiple lentigines" OR marfan*) N3 syndrome*) OR ("hepatic hypoplasia" or "arteriohepatic dysplasia*" or "bicuspid aortic valve") OR (taussig* N2 anomal*) OR ((pulmon* or aortic or subaortic or valve or mitral) N1 stenosis) OR ((aortic or aorta*) N3 coarctation*) OR (ventricular N2 dysplasia*) OR ("cor triatriatum" or cortriatriatum or "triatrial heart*") OR ("myocardial bridging*" or "crisscross heart*" or "criss-cross heart*") OR (dextrocardia* or "kartagener* triad" or "primary ciliary dyskinesia") OR ("patent ductus arteriosus" or "anomalous pulmonary venous connection" or "double inlet left ventricle" or "double outlet right ventricle" or "interrupted aortic arch") OR ("ebstein* anomaly" or "ebstein* malformation*" or

"ectopia cordis") OR (eisenmenger* N1 (complex or syndrome)) OR ("persistent truncus arteriosus" or "persistent ostium primum") OR ("endocardial cushion defect*" or "atrioventricular canal") OR ("foramen oval*") OR (heart N3 hypoplas*) OR ((noncompaction OR "non compaction") N3 "ventricular myocardium") OR (levocardia) OR (((tetralogy or trilogy or syndrome) N2 fallot*) or cantrell* or shon?s) OR ((tricuspid OR valve OR pulmonary) N1 atresia*) or ("absent right atrioventricular connection" OR "single ventricle physiology" or GUCH or "cavopulmonary connection") OR ((bonnevie N2 (syndrome* or status)) or "polynesian bronchiectas*")) OR AB (((congenital* OR hereditary OR inborn) AND ((heart* or cardiac* or coronary or septal* or aortopulmonary or aorticopulmonary or atrial or ventricular or intraventricular) N3 (defect* or disease* OR malformation* or abnormal* or anomal*))) OR (digeorge N1 (syndrome* or anomal* or sequenc*)) OR (transpos* N3 (arteries or artery or vessel*)) OR (alagille N2 syndrome) OR ("arteriohepatic dysplasia*" or "gonadal dysgenesis" or "subdivided left atrium*") OR ((cardiovertebral OR "pharyngeal pouch" OR "thymic aplasia" OR "conotruncal anomaly face"

OR turner* OR noonan OR barth OR velo* OR kartagener* OR siewert* OR scimitar OR lutembacher* OR leopard or "multiple lentigines" OR marfan*) N3 syndrome*) OR ("hepatic hypoplasia" or "arteriohepatic dysplasia*" or "bicuspid aortic valve") OR (taussig* N2 anomal*) OR ((pulmon* or aortic or subaortic or valve or mitral) N1 stenosis) OR ((aortic or aorta*) N3 coarctation*) OR (ventricular N2 dysplasia*) OR ("cor triatriatum" or cortriatriatum or "triatrial heart*") OR ("myocardial bridging*" or "crisscross heart*" or "criss-cross heart*") OR (dextrocardia* or "kartagener* triad" or "primary ciliary dyskinesia") OR ("patent ductus arteriosus" or "anomalous pulmonary venous connection" or "double inlet left ventricle" or "double outlet right ventricle" or "interrupted aortic arch") OR ("ebstein* anomaly" or "ebstein* malformation*" or "ectopia cordis") OR (eisenmenger* N1 (complex or syndrome)) OR ("persistent truncus arteriosus" or "persistent ostium primum") OR ("endocardial cushion defect*" or "atrioventricular canal") OR ("foramen oval*") OR (heart N3 hypoplas*) OR ((noncompaction OR "non compaction") N3 "ventricular myocardium") OR (levocardia) OR (((tetralogy or trilogy or syndrome) N2 fallot*) or cantrell* or shon?

s) OR ((tricuspid OR valve OR pulmonary) N1 atresia*) or ("absent right atrioventricular connection" OR "single ventricle physiology" or GUCH or "cavopulmonary connection") OR ((bonnevie N2 (syndrome* or status)) or "polynesian bronchiectas*"))

Suchprotokoll zur Studie Early motor interventions in children with CHD
Datenbank: *PEDRO*

**Suche in Pedro**	"congenital heart" > 30 Treffer in PEDRO<br>➢ Alle nach EndNote exportiert
**Suche in EN**	infant* OR baby OR babies OR neonat* OR newborn OR preterm OR prematur* OR "after birth"

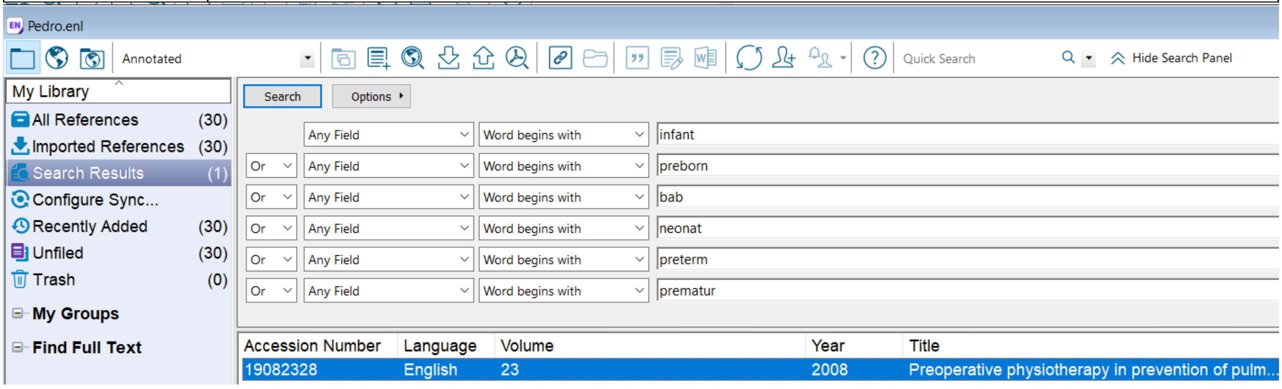

Liefert 1 Treffer

("arteriohepatic dysplasia*" or "gonadal dysgenesis" or "subdivided left atrium*") OR ((cardiovertebral OR "pharyngeal pouch" OR "thymic aplasia" OR "conotruncal anomaly face" OR turner* OR noonan OR barth OR velo* OR kartagener* OR siewert* OR scimitar OR lutembacher* OR leopard or "multiple lentigines" OR marfan*) N3 syndrome*) OR ("hepatic hypoplasia" or "arteriohepatic dysplasia*" or "bicuspid aortic valve") OR (taussig* N2 anomal*) OR ((pulmon* or aortic or subaortic or valve or mitral) N1 stenosis) OR ((aortic or aorta*) N3 coarctation*) OR (ventricular N2 dysplasia*) OR ("cor triatriatum" or cortriatriatum or "triatrial heart*") OR ("myocardial bridging*" or "crisscross heart*" or "criss-cross heart*") OR (dextrocardia* or "kartagener* triad" or "primary ciliary dyskinesia") OR ("patent ductus arteriosus" or "anomalous pulmonary venous connection" or "double inlet left ventricle" or "double outlet right ventricle" or "interrupted aortic arch") OR ("ebstein* anomaly" or "ebstein* malformation*" or "ectopia cordis") OR (eisenmenger* N1 (complex or syndrome)) OR ("persistent truncus arteriosus" or "persistent ostium primum") OR ("endocardial cushion defect*" or "atrioventricular canal") OR ("foramen oval*")

OR (heart N3 hypoplas*) OR ((noncompaction OR "non compaction") N3 "ventricular myocardium") OR (levocardia) OR (((tetralogy or trilogy or syndrome) N2 fallot*) or cantrell* or shon?s) OR ((tricuspid OR valve OR pulmonary) N1 atresia*) or ("absent right atrioventricular connection" OR "single ventricle physiology" or GUCH or "cavopulmonary connection") OR ((bonnevie N2 (syndrome* or status)) or "polynesian bronchiectas*"))
