# Supplementary material for: A systematic review of early motor interventions for infants with congenital heart disease and open-heart surgery
Source: Syst Rev. 2023 Aug 25;12:149. doi: 10.1186/s13643-023-02320-3 (PMC10463862; doi:10.1186/s13643-023-02320-3)
Supplement: Supplementary file 2 — Additional file 2. Update search strategy. [file 13643_2023_2320_MOESM2_ESM.pdf]

Suchprotokoll zur Studie Early motor interventions in children with CHD

Update-Suche: 22.04.2023
Suchprotokolle:

- DOKU_CINAHL_EBSCOhost_mgoste.pdf
- DOKU_Cochrane_mgoste.pdf
- DOKU_DB_Strategies_mgoste.xlsx
- DOKU_EMBASE_mgoste.pdf
- DOKU_Medline_EBSCOhost_mgoste.pdf
- DOKU_Pedro_mgoste.pdf
- DOKU_PsycINFO_EBSCOhost_mgoste.pdf
- DOKU_Scopus_mgoste.pdf

	**Deduplication**	
	Before	after
Medline	23	23
EMBASE	102	90
CINAHL	15	6
Cochrane	12	7
PsycINFO (dedoubliert gegen Search 2020 in EN, siehe S.2)	2	2
PEDRO	1	1
Scopus	62	28
Pool	217	**157***

*automatisch dedoubliert, kann noch einzelne Duplikate enthalten

Reference files: Update_23_Early motor interventions in children with CHD.enlx

Before:

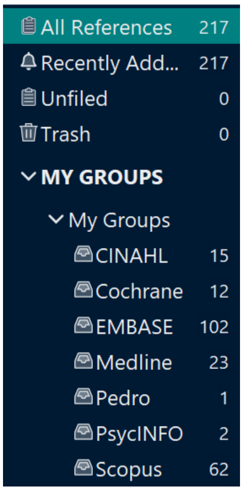

after:

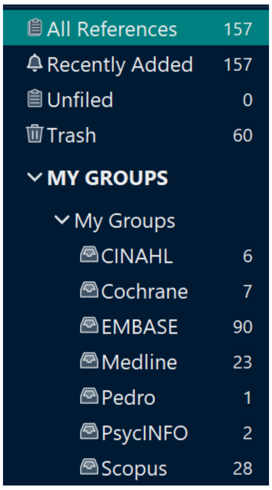

PsycInfo-Deduplication

Before **after:**

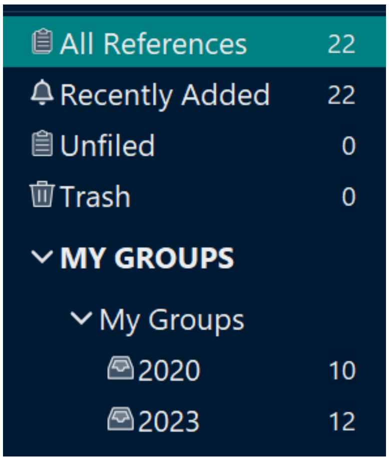

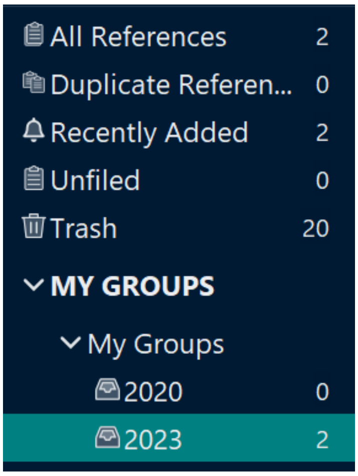

EBSCOhost

Saturday, April 22, 2023 8:04:33 AM

#	Query	Limiters/Expanders	Last Run Via	Results
S8	(S1 AND S2 AND S3 AND S4) AND EM 20200512-	Limiters - Language: English, French, German, Italian<br>Expanders - Apply equivalent subjects<br>Search modes - Find all my search terms	Interface - EBSCOhost Research Databases<br>Search Screen - Advanced Search<br>Database - CINAHL with Full Text	15
S7	(S1 AND S2 AND S3 AND S4) AND EM 20200512-	Expanders - Apply equivalent subjects<br>Search modes - Find all my search terms	Interface - EBSCOhost Research Databases<br>Search Screen - Advanced Search<br>Database - CINAHL with Full Text	15
S6	S1 AND S2 AND S3 AND S4	Limiters - Language: English, French, German, Italian<br>Expanders - Apply equivalent subjects<br>Search modes - Find all my search terms	Interface - EBSCOhost Research Databases<br>Search Screen - Advanced Search<br>Database - CINAHL with Full Text	60
S5	S1 AND S2 AND S3 AND S4	Expanders - Apply equivalent subjects<br>Search modes - Find all my search terms	Interface - EBSCOhost Research Databases<br>Search Screen - Advanced Search<br>Database - CINAHL with Full Text	62
S4	(MH "Treatment Outcomes+") OR (MH "Outcome Assessment") OR (MH "Upper Extremity+") OR (MH "Lower Extremity+") OR (MH "Motor Skills+") OR (MH "Motor Skills Disorders") OR (MH "Gait Disorders, Neurologic+") OR TI	Expanders - Apply equivalent subjects<br>Search modes - Find all my search terms	Interface - EBSCOhost Research Databases<br>Search Screen - Advanced Search<br>Database - CINAHL with Full Text	1,292,526

	(motor N3 (skill* OR ability* OR develop* OR outcome* OR function* OR gross OR fine OR activit* OR performance OR improve*)) OR TI ((limb OR extremit* OR hand OR foot) N3 (function OR skill* OR ability*)) OR TI (outcome OR movement OR gait) OR AB (motor N3 (skill* OR ability* OR develop* OR outcome* OR function* OR gross OR fine OR activit* OR performance OR improve*)) OR AB ((limb OR extremit* OR hand OR foot) N3 (function OR skill* OR ability*)) OR AB (outcome OR movement OR gait)			
S3	(MH "Heart Defects, Congenital+/RH") OR (MH "Physical Therapy+") OR (MH "Pediatric Physical Therapy") OR (MH "Physical Therapists") OR (MH "Therapeutic Exercise+") OR (MH "Restraint, Physical") OR (MH "Early Childhood Intervention") OR (MH "Occupational Therapy") OR (MH "Pediatric Occupational Therapy") OR (MH "Occupational Therapists") OR TI ((physical OR occupational OR constraint-induced OR neurodevelopment* OR neuro-development* OR	Expanders - Apply equivalent subjects<br>Search modes - Find all my search terms	Interface - EBSCOhost Research Databases<br>Search Screen - Advanced Search<br>Database - CINAHL with Full Text	276,216

	motor OR movement OR exercise) N3 (train* OR therap* OR intervention* OR treat* OR support* OR enhance*)) OR TI (physiotherap* OR NDT OR bobath* OR Vojta*) OR AB ((physical OR occupational OR constraint-induced OR neurodevelopment* OR neuro-development* OR motor OR movement OR exercise) N3 (train* OR therap* OR intervention* OR treat* OR support* OR enhance*)) OR AB (physiotherap* OR NDT OR bobath* OR Vojta*)			
S2	(MH "Pediatrics+") OR (MH "Infant+") OR (MH "Intensive Care Units, Pediatric+") OR TI (infant* OR baby OR babies OR neonat* OR newborn OR preterm OR prematur* OR "after birth") OR AB (infant* OR baby OR babies OR neonat* OR newborn OR preterm OR prematur* OR "after birth")	Expanders - Apply equivalent subjects<br>Search modes - Find all my search terms	Interface - EBSCOhost Research Databases<br>Search Screen - Advanced Search<br>Database - CINAHL with Full Text	424,184
S1	(MH "Heart Defects, Congenital+") OR TI (((congenital* OR hereditary OR inborn) AND ((heart* or cardiac* or coronary or septal* or aortopulmonary or aorticopulmonary or atrial or ventricular or intraventricular) N3	Expanders - Apply equivalent subjects<br>Search modes - Find all my search terms	Interface - EBSCOhost Research Databases<br>Search Screen - Advanced Search<br>Database - CINAHL with Full Text	46,592

(defect* or disease* OR malformation* or abnormal* or anomal*))) OR (digeorge N1 (syndrome* or anomal* or sequenc*)) OR (transpos* N3 (arteries or artery or vessel*)) OR (alagille N2 syndrome) OR ("arteriohepatic dysplasia*" or "gonadal dysgenesis" or "subdivided left atrium*") OR ((cardiovertebral OR "pharyngeal pouch" OR "thymic aplasia" OR "conotruncal anomaly face" OR turner* OR noonan OR barth OR velo* OR kartagener* OR siewert* OR scimitar OR lutembacher* OR leopard or "multiple lentigines" OR marfan*) N3 syndrome*) OR ("hepatic hypoplasia" or "arteriohepatic dysplasia*" or "bicuspid aortic valve") OR (taussig* N2 anomal*) OR ((pulmon* or aortic or subaortic or valve or mitral) N1 stenosis) OR ((aortic or aorta*) N3 coarctation*) OR (ventricular N2 dysplasia*) OR ("cor triatriatum" or cortriatriatum or "triatrial heart*") OR ("myocardial bridging*" or "crisscross heart*" or "criss-cross heart*") OR (dextrocardia* or "kartagener* triad" or "primary ciliary

dyskinesia") OR ("patent ductus arteriosus" or "anomalous pulmonary venous connection" or "double inlet left ventricle" or "double outlet right ventricle" or "interrupted aortic arch") OR ("ebstein* anomaly" or "ebstein* malformation*" or "ectopia cordis") OR (eisenmenger* N1 (complex or syndrome)) OR ("persistent truncus arteriosus" or "persistent ostium primum") OR ("endocardial cushion defect*" or "atrioventricular canal") OR ("foramen oval*") OR (heart N3 hypoplas*) OR ((noncompaction OR "non compaction") N3 "ventricular myocardium") OR (levocardia) OR (((tetralogy or trilogy or syndrome) N2 fallot*) or cantrell* or shon?s) OR ((tricuspid OR valve OR pulmonary) N1 atresia*) or ("absent right atrioventricular connection" OR "single ventricle physiology" or GUCH or "cavopulmonary connection") OR ((bonnevie N2 (syndrome* or status)) or "polynesian bronchiectas*")) OR AB (((congenital* OR hereditary OR inborn) AND ((heart* or cardiac*

or coronary or septal* or aortopulmonary or aorticopulmonary or atrial or ventricular or intraventricular) N3 (defect* or disease* OR malformation* or abnormal* or anomal*))) OR (digeorge N1 (syndrome* or anomal* or sequenc*)) OR (transpos* N3 (arteries or artery or vessel*)) OR (alagille N2 syndrome) OR ("arteriohepatic dysplasia*" or "gonadal dysgenesis" or "subdivided left atrium*") OR ((cardiovertebral OR "pharyngeal pouch" OR "thymic aplasia" OR "conotruncal anomaly face" OR turner* OR noonan OR barth OR velo* OR kartagener* OR siewert* OR scimitar OR lutembacher* OR leopard or "multiple lentigines" OR marfan*) N3 syndrome*) OR ("hepatic hypoplasia" or "arteriohepatic dysplasia*" or "bicuspid aortic valve") OR (taussig* N2 anomal*) OR ((pulmon* or aortic or subaortic or valve or mitral) N1 stenosis) OR ((aortic or aorta*) N3 coarctation*) OR (ventricular N2 dysplasia*) OR ("cor triatriatum" or cortriatriatum or "triatrial heart*") OR ("myocardial bridging*" or "crisscross

heart*" or "criss-cross heart*") OR (dextrocardia* or "kartagener* triad" or "primary ciliary dyskinesia") OR ("patent ductus arteriosus" or "anomalous pulmonary venous connection" or "double inlet left ventricle" or "double outlet right ventricle" or "interrupted aortic arch") OR ("ebstein* anomaly" or "ebstein* malformation*" or "ectopia cordis") OR (eisenmenger* N1 (complex or syndrome)) OR ("persistent truncus arteriosus" or "persistent ostium primum") OR ("endocardial cushion defect*" or "atrioventricular canal") OR ("foramen oval*") OR (heart N3 hypoplas*) OR ((noncompaction OR "non compaction") N3 "ventricular myocardium") OR (levocardia) OR (((tetralogy or trilogy or syndrome) N2 fallot*) or cantrell* or shon?s) OR ((tricuspid OR valve OR pulmonary) N1 atresia*) or ("absent right atrioventricular connection" OR "single ventricle physiology" or GUCH or "cavopulmonary connection") OR ((bonnevie N2 (syndrome* or status))

or "polynesian
bronchiectas*"))

Cochrane Library

Access provided by: **University Library Zurich / Zentralbibliothek Zürich**

We noticed your browser language is German.
You can select your preferred language at the top of any page, and you will see translated Cochrane Review sections in this language. Change to **German.**

Advanced Search

Search manager

Save this search | View/Share saved searches | ? Search help

Print search history

#1

((congenital* OR hereditary OR inborn):ti,ab,kw AND ((heart* or cardiac* or coronary or septal* or aortopulmonary or aorticopulmonary or atrial or ventricular or intraventricular) NEAR/3 (defect* or disease* OR malformation* or abnormal* or anomal*)):ti,ab,kw) OR (digeorge NEAR/1 (syndrome* or anomal* or sequenc*)):ti,ab,kw OR (transpos* NEAR/3 (arteries or artery or vessel*)):ti,ab,kw OR (alagille NEAR/2 syndrome):ti,ab,kw OR ("arteriohepatic dysplasia*" or "gonadal dysgenesis" or "subdivided left atrium*"):ti,ab,kw OR ((cardiovertebral OR "pharyngeal pouch" OR "thymic aplasia" OR "conotruncal anomaly face" OR turner* OR noonan OR barth OR velo* OR kartagener* OR siewert* OR scimitar OR lutembacher* OR leopard or "multiple lentigines" OR marfan*) NEAR/3 syndrome*):ti,ab,kw OR ("hepatic hypoplasia" or "arteriohepatic dysplasia*" or "bicuspid aortic valve"):ti,ab,kw OR (taussig* NEAR/2 anomal*):ti,ab,kw OR ((pulmon* or aortic or subaortic or valve or mitral) NEAR/1 stenosis):ti,ab,kw OR ((aortic or aorta*) NEAR/3 coarctation*):ti,ab,kw OR (ventricular NEAR/2 dysplasia*):ti,ab,kw OR ("cor triatriatum" or cortriatriatum or "triatrial heart*"):ti,ab,kw OR ("myocardial bridging*" or "crisscross heart*" or "criss-cross heart*"):ti,ab,kw OR (dextrocardia* or "kartagener* triad" or "primary ciliary dyskinesia"):ti,ab,kw OR ("patent ductus arteriosus" or "anomalous pulmonary venous connection" or "double inlet left ventricle" or "double outlet right ventricle" or "interrupted aortic arch"):ti,ab,kw OR ("ebstein* anomaly" or "ebstein* malformation*" or "ectopia cordis"):ti,ab,kw OR (eisenmenger* NEAR/1 (complex or syndrome)):ti,ab,kw OR ("persistent truncus arteriosus" or "persistent ostium primum"):ti,ab,kw OR ("endocardial cushion defect*" or "atrioventricular canal"):ti,ab,kw OR ("foramen oval*"):ti,ab,kw OR (heart NEAR/3 hypoplas*):ti,ab,kw OR

S | MeSH | Limits | 7221

((noncompaction OR "non compaction") NEAR/3 "ventricular myocardium"):ti,ab,kw OR (levocardia):ti,ab,kw OR (((tetralogy or trilogy or syndrome) NEAR/2 fallot*) or cantrell* or shon?s):ti,ab,kw OR ((tricuspid OR valve OR pulmonary) NEAR/1 atresia*):ti,ab,kw or ("absent right atrioventricular connection" OR "single ventricle physiology" or GUCH or "cavopulmonary connection"):ti,ab,kw OR ((bonnevie NEAR/2 (syndrome* or status)) or "polynesian bronchiectas*"):ti,ab,kw

#2	(infant* OR baby OR babies OR neonat* OR newborn OR preterm OR prematur* OR "after birth"):ti,ab,kw	Limits	101685
#3	((physical OR occupational OR constraint-induced OR neurodevelopment* OR neuro-development* OR motor OR movement OR exercise) NEAR/3 (train* OR therap* OR intervention* OR treat* OR support* OR enhance*)):ti,ab,kw OR (physiotherap* OR NDT OR [illegible]	Limits	88517
#4	(motor NEAR/3 (skill* OR ability* OR develop* OR outcome* OR function* OR gross OR fine OR activit* OR performance OR improve*)):ti,ab,kw OR ((limb OR extremit* OR hand OR foot) NEAR/3 (function OR skill* OR ability*)):ti,ab,kw OR (outcome OR [illegible]	Limits	668935
#5	#1 AND #2 AND #3 AND #4	Limits	31
#6	#1 AND #2 AND #3 AND #4	Limits	12

with Publication Year from 2020 to present , with Cochrane Library publication date from May 2020 to present, in Trials

#7 Type a search term or use the S or MeSH buttons to compose | S | MeSH | Limits | N/A

Clear all

Highlight orphan lines

Save this search | View/Share saved searches | Search help

Print search history

Filter your results

Cochrane Reviews 0 | Cochrane Protocols 0 | Trials 12 | Editorials 0 | Special Collections 0 | Clinical Answers 0 | More

For COVID-19 related studies, please also see the **Cochrane COVID-19 Study Register**

12 Trials matching **"#6 - #1 AND #2 AND #3 AND #4" with Publication Year from 2020 to present, with Cochrane Library publication date from May 2020 to present, in Trials**

Cochrane Central Register of Controlled Trials
Issue 4 of 12, April 2023

Deselect all (12) Export selected citation(s)

Order by Relevancy

Results per page 25

1

Family-centered early motor intervention in infants with complex congenital heart disease: protocol for a randomized controlled pilot trial

E Mitteregger, T Dirks, M Theiler, O Kretschmar, B Latal

Cardiology in the young, **2022**, 32, S240 | added to CENTRAL: 31 October 2022 | 2022 Issue 10

Embase

2

A family-tailored early motor intervention (EMI-Heart) for infants with complex congenital heart disease: study protocol for a feasibility RCT

E Mitteregger, T Dirks, M Theiler, O Kretschmar, B Latal

Pilot and feasibility studies, **2022**, 8(1) | added to CENTRAL: 31 January 2023 | 2023 Issue 1

Embase

3

Family Centred Early Intervention of Infants With Congenital Heart Disease

NCT04666857

https://clinicaltrials.gov/show/NCT04666857, **2020** | added to CENTRAL: 31 December 2020 | 2020 Issue 12

CT.gov

4 ☑

Belly time in the development of premature newborns

RBR-2nwkr47

https://trialsearch.who.int/Trial2.aspx?TrialID=RBR-2nwkr47, **2022** | added to CENTRAL: 30 June 2022 | 2022 Issue 06

ICTRP

5 ☑

Evaluations of Cardiopulmonary Function and Motor Development of Congenital Heart Disease

NCT05518136

https://clinicaltrials.gov/show/NCT05518136, **2022** | added to CENTRAL: 30 September 2022 | 2022 Issue 9

CT.gov

6 ☑

Design and Rationale of RE-ENERGIZE FONTAN: randomizEd Exercise iNtERvention desiGned to maximIZE fitness in FONTAN patients

ESS Tierney, L Palaniappan, M Leonard, J Long, J Myers, T Davila, MC Lui, F Kogan, I Olson, R Punn, M Desai, LM Schneider, C-H Wang, JP Cooke, D Bernstein

American heart journal, **2023** | added to CENTRAL: 31 March 2023 | 2023 Issue 3

PubMed Embase

7 ☑

Evaluation of a "tummy time" intervention to improve motor skills in infants after cardiac surgery

K Uzark, C Smith, S Yu, R Lowery, C Tapley, JC Romano, J Butcher

Cardiology in the young, **2022**, 32(8), 1210-1215 | added to CENTRAL: 30 November 2021 | 2021 Issue 11
PubMed Embase

8 ☑

A Feasibility Study of Physical Activity After Surgical or Catheterization Intervention

NCT04619745

https://clinicaltrials.gov/show/NCT04619745, **2020** | added to CENTRAL: 30 November 2020 | 2020 Issue 11
CT.gov

9 ☑

Association between Term Equivalent Brain Magnetic Resonance Imaging and 2-Year Outcomes in Extremely Preterm Infants: a Report from the Preterm Erythropoietin Neuroprotection Trial Cohort

DE Mayock, S Gogcu, M Puia-Dumitrescu, DWW Shaw, JN Wright, BA Comstock, PJ Heagerty, SE Juul

Journal of pediatrics, **2021**, 239, 117-125.e6 | added to CENTRAL: 31 December 2021 | 2021 Issue 12
PubMed Embase

10 ☑

A randomised placebo-controlled trial of the effectiveness of early metformin in addition to usual care in the reduction of gestational diabetes mellitus effects (EMERGE): study protocol

F Dunne, C Newman, D Devane, A Smyth, A Alvarez-Iglesias, P Gillespie, M Browne, M O'Donnell

Trials, **2022**, 23(1), 795 | added to CENTRAL: 31 October 2022 | 2022 Issue 10
PubMed Embase

11 ☑

Design and methods for the training in exercise activities and motion for growth (TEAM 4 growth) trial: a randomized controlled trial

LM Lambert, VL Pemberton, FL Trachtenberg, K Uzark, F Woodard, JE Teng, J Bainton, S Clarke, L Justice, MR Meador, J Riggins, M Suhre, D Sylvester, S Butler, TA Miller

International journal of cardiology, **2022**, 359, 28-34 | added to CENTRAL: 31 May 2022 | 2022 Issue 05

PubMed Embase

12 ☑

Randomized Controlled Trial of Working Memory Intervention in Congenital Heart Disease

J Calderon, D Wypij, V Rofeberg, C Stopp, A Roseman, D Albers, JW Newburger, DC Bellinger

Journal of pediatrics, **2020**, 227, 191-198.e3 | added to CENTRAL: 30 September 2020 | 2020 Issue 09

PubMed Embase

Embase

Embase session results (22 Apr 2023)

No.	Query	Results
#7	#1 AND #2 AND #3 AND #4 AND ([english]/lim OR [french]/lim OR [german]/lim OR [italian]/lim) AND [12-05-2020]/sd	102
#6	#1 AND #2 AND #3 AND #4 AND ([english]/lim OR [french]/lim OR [german]/lim OR [italian]/lim)	345
#5	#1 AND #2 AND #3 AND #4	346
#4	'treatment outcome'/exp OR 'outcome assessment'/exp OR 'upper limb'/exp OR 'lower limb'/exp OR 'psychomotor disorder'/exp OR 'neurologic gait disorder'/exp OR ((motor NEAR/3 (skill* OR ability* OR develop* OR outcome* OR function* OR gross OR fine OR activit* OR performance OR improve*)):ti,ab) OR (((limb OR extremit* OR hand OR foot) NEAR/3 (function OR skill* OR ability*)):ti,ab) OR outcome:ti,ab OR movement:ti,ab OR gait:ti,ab	4543339
#3	'congenital heart disease'/exp/dm_rh OR 'physiotherapy'/exp OR 'kinesiotherapy'/exp OR 'exercise therapy in infancy and childhood'/exp OR 'exercise'/exp OR 'physiotherapist'/exp OR 'occupational therapy'/exp OR 'occupational therapist'/exp OR 'early childhood intervention'/exp OR (((physical OR occupational OR 'constraint induced' OR neurodevelopment* OR 'neuro development*' OR motor OR movement OR exercise) NEAR/3 (train* OR therap* OR intervention* OR treat* OR support* OR enhance*)):ti,ab) OR physiotherap*:ti,ab OR ndt:ti,ab OR bobath*:ti,ab OR vojta*:ti,ab	710013
#2	'pediatrics'/exp OR 'infant'/exp OR infant*:ti,ab OR baby:ti,ab OR babies:ti,ab OR neonat*:ti,ab OR newborn:ti,ab OR preterm:ti,ab OR prematur*:ti,ab OR 'after birth':ti,ab	1957606

No.	Query	Results
#1	'congenital heart disease'/exp OR 'heart disease'/exp/dm_cn OR ((congenital*:ti,ab OR hereditary:ti,ab OR inborn:ti,ab) AND (((heart* OR cardiac* OR coronary OR septal* OR aortopulmonary OR aorticopulmonary OR atrial OR ventricular OR intraventricular) NEAR/3 (defect* OR disease* OR malformation* OR abnormal* OR anomal*)):ti,ab)) OR ((digeorge NEAR/1 (syndrome* OR anomal* OR sequenc*)):ti,ab) OR ((transpos* NEAR/3 (arteries OR artery OR vessel*)):ti,ab) OR ((alagille NEAR/2 syndrome):ti,ab) OR 'gonadal dysgenesis':ti,ab OR 'subdivided left atrium*':ti,ab OR (((cardiovertebral OR 'pharyngeal pouch' OR 'thymic aplasia' OR 'conotruncal anomaly face' OR turner* OR noonan OR barth OR velo* OR kartagener* OR siewert* OR scimitar OR lutembacher* OR leopard OR 'multiple lentigines' OR marfan*) NEAR/3 syndrome*):ti,ab) OR 'hepatic hypoplasia':ti,ab OR 'arteriohepatic dysplasia*':ti,ab OR 'bicuspid aortic valve':ti,ab OR ((taussig* NEAR/2 anomal*):ti,ab) OR (((pulmon* OR aortic OR subaortic OR valve OR mitral) NEAR/1 stenosis):ti,ab) OR (((aortic OR aorta*) NEAR/3 coarctation*):ti,ab) OR ((ventricular NEAR/2 dysplasia*):ti,ab) OR 'cor triatriatum':ti,ab OR cortriatriatum:ti,ab OR 'triatrial heart*':ti,ab OR 'myocardial bridging*':ti,ab OR 'crisscross heart*':ti,ab OR 'criss-cross heart*':ti,ab OR dextrocardia*:ti,ab OR 'kartagener* triad':ti,ab OR 'primary ciliary dyskinesia':ti,ab OR 'patent ductus arteriosus':ti,ab OR 'anomalous pulmonary venous connection':ti,ab OR 'double inlet left ventricle':ti,ab OR 'double outlet right ventricle':ti,ab OR 'interrupted aortic arch':ti,ab OR 'ebstein* anomaly':ti,ab OR 'ebstein* malformation*':ti,ab OR 'ectopia cordis':ti,ab OR ((eisenmenger* NEAR/1 (complex OR syndrome)):ti,ab) OR 'persistent truncus arteriosus':ti,ab OR 'persistent ostium primum':ti,ab OR 'endocardial cushion defect*':ti,ab OR 'atrioventricular canal':ti,ab OR 'foramen oval*':ti,ab OR ((heart NEAR/3 hypoplas*):ti,ab) OR (((noncompaction OR 'non compaction') NEAR/3 'ventricular myocardium'):ti,ab) OR levocardia:ti,ab OR (((tetralogy OR trilogy OR syndrome) NEAR/2 fallot*):ti,ab) OR cantrell*:ti,ab OR shon?s:ti,ab OR (((tricuspid OR valve OR pulmonary) NEAR/1 atresia*):ti,ab) OR 'absent right atrioventricular connection':ti,ab OR 'single ventricle physiology':ti,ab OR guch:ti,ab OR 'cavopulmonary connection':ti,ab OR ((bonnevie NEAR/2 (syndrome* OR status)):ti,ab) OR 'polynesian bronchiectas*':ti,ab	336224

Copyright © 2023 Elsevier Limited except certain content provided by third parties.

Embase is a trade mark of Elsevier Life Sciences IP Limited.

Saturday, April 22, 2023 7:55:36 AM

#	Query	Limiters/Expanders	Last Run Via	Results
S7	(S1 AND S2 AND S3 AND S4) AND EM 20200512-	Expanders - Apply equivalent subjects<br>Search modes - Find all my search terms	Interface - EBSCOhost Research Databases<br>Search Screen - Advanced Search<br>Database - MEDLINE	23
S6	S1 AND S2 AND S3 AND S4	Limiters - Language: English, French, German, Italian<br>Expanders - Apply equivalent subjects<br>Search modes - Find all my search terms	Interface - EBSCOhost Research Databases<br>Search Screen - Advanced Search<br>Database - MEDLINE	137
S5	S1 AND S2 AND S3 AND S4	Expanders - Apply equivalent subjects<br>Search modes - Find all my search terms	Interface - EBSCOhost Research Databases<br>Search Screen - Advanced Search<br>Database - MEDLINE	140
S4	(MH "Treatment Outcome+") OR (MH "Upper Extremity") OR (MH "Lower Extremity+") OR (MH "Motor Skills") OR (MH "Motor Skills Disorders") OR (MH "Gait Disorders, Neurologic+") OR TI (motor N3 (skill* OR ability* OR develop* OR outcome* OR function* OR gross OR fine OR activit* OR performance OR improve*)) OR TI ((limb OR extremit* OR hand OR foot) N3 (function OR skill* OR ability*)) OR TI (outcome OR movement OR gait) OR AB (motor	Expanders - Apply equivalent subjects<br>Search modes - Find all my search terms	Interface - EBSCOhost Research Databases<br>Search Screen - Advanced Search<br>Database - MEDLINE	3,551,089

	N3 (skill* OR ability* OR develop* OR outcome* OR function* OR gross OR fine OR activit* OR performance OR improve*)) OR AB ((limb OR extremit* OR hand OR foot) N3 (function OR skill* OR ability*)) OR AB (outcome OR movement OR gait)			
S3	(MH "Heart Defects, Congenital+/RH") OR (MH "Physical Therapy Modalities+") OR (MH "Physical Therapy Specialty") OR (MH "Exercise Therapy+") OR (MH "Physical Therapists") OR (MH "Occupational Therapists") OR (MH "Occupational Therapy") OR (MH "Restraint, Physical+") OR (MH "Early Intervention, Educational") OR TI ((physical OR occupational OR constraint-induced OR neurodevelopment* OR neuro-development* OR motor OR movement OR exercise) N3 (train* OR therap* OR intervention* OR treat* OR support* OR enhance*)) OR TI (physiotherap* OR NDT OR bobath* OR Vojta*) OR AB ((physical OR occupational OR constraint-induced OR neurodevelopment* OR neuro-development* OR motor OR movement	Expanders - Apply equivalent subjects<br>Search modes - Find all my search terms	Interface - EBSCOhost Research Databases<br>Search Screen - Advanced Search<br>Database - MEDLINE	362,741

	OR exercise) N3 (train* OR therap* OR intervention* OR treat* OR support* OR enhance*)) OR AB (physiotherap* OR NDT OR bobath* OR Vojta*)			
S2	(MH "Pediatrics+") OR (MH "Infant") OR (MH "Intensive Care Units, Pediatric+") OR TI (infant* OR baby OR babies OR neonat* OR newborn OR preterm OR prematur* OR "after birth") OR AB (infant* OR baby OR babies OR neonat* OR newborn OR preterm OR prematur* OR "after birth")	Expanders - Apply equivalent subjects<br>Search modes - Find all my search terms	Interface - EBSCOhost Research Databases<br>Search Screen - Advanced Search<br>Database - MEDLINE	1,605,997
S1	(MH "Heart Defects, Congenital+") OR (MH "Heart Diseases+/CN") OR TI (((congenital* OR hereditary OR inborn) AND ((heart* or cardiac* or coronary or septal* or aortopulmonary or aorticopulmonary or atrial or ventricular or intraventricular) N3 (defect* or disease* OR malformation* or abnormal* or anomal*))) OR (digeorge N1 (syndrome* or anomal* or sequenc*)) OR (transpos* N3 (arteries or artery or vessel*)) OR (alagille N2 syndrome) OR ("arteriohepatic dysplasia*" or "gonadal dysgenesis" or "subdivided left atrium*")	Expanders - Apply equivalent subjects<br>Search modes - Find all my search terms	Interface - EBSCOhost Research Databases<br>Search Screen - Advanced Search<br>Database - MEDLINE	249,856

OR ((cardiovertebral OR "pharyngeal pouch" OR "thymic aplasia" OR "conotruncal anomaly face" OR turner* OR noonan OR barth OR velo* OR kartagener* OR siewert* OR scimitar OR lutembacher* OR leopard or "multiple lentigines" OR marfan*) N3 syndrome*) OR ("hepatic hypoplasia" or "arteriohepatic dysplasia*" or "bicuspid aortic valve") OR (taussig* N2 anomal*) OR ((pulmon* or aortic or subaortic or valve or mitral) N1 stenosis) OR ((aortic or aorta*) N3 coarctation*) OR (ventricular N2 dysplasia*) OR ("cor triatriatum" or cortriatriatum or "triatrial heart*") OR ("myocardial bridging*" or "crisscross heart*" or "criss-cross heart*") OR (dextrocardia* or "kartagener* triad" or "primary ciliary dyskinesia") OR ("patent ductus arteriosus" or "anomalous pulmonary venous connection" or "double inlet left ventricle" or "double outlet right ventricle" or "interrupted aortic arch") OR ("ebstein* anomaly" or "ebstein* malformation*" or "ectopia cordis") OR (eisenmenger* N1

(complex or syndrome)) OR ("persistent truncus arteriosus" or "persistent ostium primum") OR ("endocardial cushion defect*" or "atrioventricular canal") OR ("foramen oval*") OR (heart N3 hypoplas*) OR ((noncompaction OR "non compaction") N3 "ventricular myocardium") OR (levocardia) OR (((tetralogy or trilogy or syndrome) N2 fallot*) or cantrell* or shon?s) OR ((tricuspid OR valve OR pulmonary) N1 atresia*) or ("absent right atrioventricular connection" OR "single ventricle physiology" or GUCH or "cavopulmonary connection") OR ((bonnevie N2 (syndrome* or status)) or "polynesian bronchiectas*")) OR AB (((congenital* OR hereditary OR inborn) AND ((heart* or cardiac* or coronary or septal* or aortopulmonary or aorticopulmonary or atrial or ventricular or intraventricular) N3 (defect* or disease* OR malformation* or abnormal* or anomal*))) OR (digeorge N1 (syndrome* or anomal* or sequenc*)) OR (transpos* N3 (arteries or artery or vessel*)) OR

(alagille N2 syndrome) OR ("arteriohepatic dysplasia*" or "gonadal dysgenesis" or "subdivided left atrium*") OR ((cardiovertebral OR "pharyngeal pouch" OR "thymic aplasia" OR "conotruncal anomaly face" OR turner* OR noonan OR barth OR velo* OR kartagener* OR siewert* OR scimitar OR lutembacher* OR leopard or "multiple lentigines" OR marfan*) N3 syndrome*) OR ("hepatic hypoplasia" or "arteriohepatic dysplasia*" or "bicuspid aortic valve") OR (taussig* N2 anomal*) OR ((pulmon* or aortic or subaortic or valve or mitral) N1 stenosis) OR ((aortic or aorta*) N3 coarctation*) OR (ventricular N2 dysplasia*) OR ("cor triatriatum" or cortriatriatum or "triatrial heart*") OR ("myocardial bridging*" or "crisscross heart*" or "criss-cross heart*") OR (dextrocardia* or "kartagener* triad" or "primary ciliary dyskinesia") OR ("patent ductus arteriosus" or "anomalous pulmonary venous connection" or "double inlet left ventricle" or "double outlet right ventricle" or "interrupted aortic arch")

OR ("ebstein* anomaly" or "ebstein* malformation*" or "ectopia cordis") OR (eisenmenger* N1 (complex or syndrome)) OR ("persistent truncus arteriosus" or "persistent ostium primum") OR ("endocardial cushion defect*" or "atrioventricular canal") OR ("foramen oval*") OR (heart N3 hypoplas*) OR ((noncompaction OR "non compaction") N3 "ventricular myocardium") OR (levocardia) OR (((tetralogy or trilogy or syndrome) N2 fallot*) or cantrell* or shon?s) OR ((tricuspid OR valve OR pulmonary) N1 atresia*) or ("absent right atrioventricular connection" OR "single ventricle physiology" or GUCH or "cavopulmonary connection") OR ((bonnevie N2 (syndrome* or status)) or "polynesian bronchiectas*"))

Suchprotokoll zur Studie Early motor interventions in children with CHD
Datenbank: *PEDRO*

**Suche in Pedro**	"congenital heart" > 40 Treffer in PEDRO<br>➢ Alle nach EndNote exportiert<br>➢ Noch 1 Treffer
**Suche in EN**	infant* OR baby OR babies OR neonat* OR newborn OR preterm OR prematur* OR "after birth"

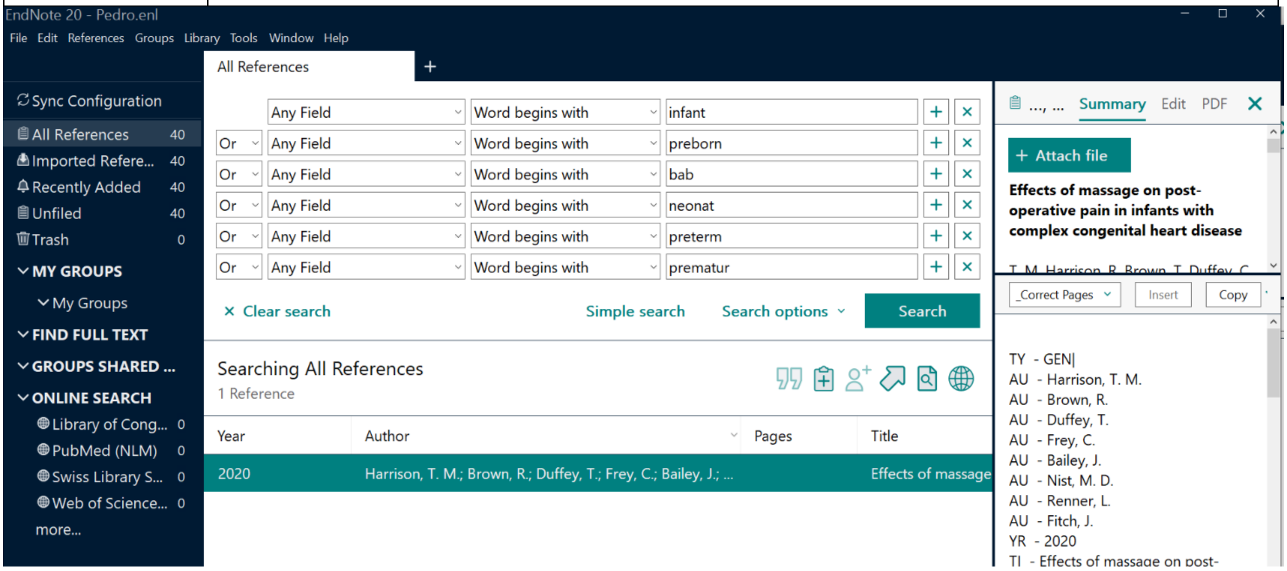

Liefert 1 Treffer

Brought to you by UZH Hauptbibliothek / Zentralbibliothek Zürich

Scopus

Advanced search

< Basic Search

Advanced

Search

Enter query string

Outline query

Add Author name / Affiliation

Sear

ALL("Cognitive architectures") AND AUTHOR-NAME(smith)
TITLE-ABS-KEY(*somatic complaint wom?n) AND PUBYEAR AFT 1993
SRCTITLE(*field ornith*) AND VOLUME(75) AND ISSUE(1) AND PAGES(53-66)

Search history

View Less

(TITLE-ABS-KEY (((congenital* OR hereditary OR inborn) AND ((heart* OR cardiac* OR coronary OR septal* OR aortopulmonary OR aorticopulmonary OR atrial OR ventricular OR intraventricular) W/3 (defect* OR disease* OR malformation* OR abnormal* OR anomal*))) OR (digeorge W/1 (syndrome* OR anomal* OR sequenc*)) OR (transpos* W/3 (arteries OR artery OR vessel*)) OR (alagille W/2 syndrome) OR ("arteriohepatic dysplasia*" OR "gonadal dysgenesis" OR "subdivided left atrium*") OR ((cardiovertebral OR "pharyngeal pouch" OR "thymic aplasia" OR "conotruncal anomaly face" OR turner* OR noonan OR barth OR velo* OR kartagener* OR siewert* OR scimitar OR lutembacher* OR leopard OR "multiple lentigines" OR marfan*) W/3 syndrome*) OR ("hepatic hypoplasia" OR "arteriohepatic dysplasia*" OR "bicuspid aortic valve") OR (taussig* W/2 anomal*) OR ((pulmon* OR aortic OR subaortic OR valve OR mitral) W/1 stenosis) OR ((aortic OR aorta*) W/3 coarctation*) OR (ventricular W/2 dysplasia*) OR ("cor triatriatum" OR cortriatriatum OR "triatrial heart*") OR ("myocardial bridging*" OR "crisscross heart*" OR "criss-cross heart*") OR (dextrocardia* OR "kartagener* triad" OR "primary ciliary dyskinesia") OR ("patent ductus arteriosus" OR "anomalous pulmonary venous connection" OR "double inlet left ventricle" OR "double outlet right ventricle" OR "interrupted aortic arch") OR ("ebstein* anomaly" OR "ebstein* malformation*" OR
4 "ectopia cordis") OR (eisenmenger* W/1 (complex OR syndrome)) OR ("persistent truncus arteriosus" OR "persistent ostium primum") OR ("endocardial cushion defect*" OR "atrioventricular canal") OR ("foramen oval*") OR (heart W/3 hypoplas*) OR ((noncompaction OR "non compaction") W/3 "ventricular myocardium") OR (levocardia) OR (((tetralogy OR trilogy OR syndrome) W/2 fallot*) OR cantrell* OR shon?s) OR ((tricuspid OR valve OR pulmonary) W/1 atresia*) OR ("absent right atrioventricular connection" OR "single ventricle physiology" OR guch OR "cavopulmonary connection") OR ((bonnevie W/2 (syndrome* OR status)) OR "polynesian bronchiectas*"))) AND (TITLE-ABS-KEY (infant* OR baby OR babies OR neonat* OR newborn OR preterm OR prematur* OR "after birth")) AND (TITLE-ABS-KEY ((physical OR occupational OR constraint-induced OR neurodevelopment* OR neuro-development* OR motor OR movement OR exercise) W/3 (train* OR therap* OR intervention* OR treat* OR support* OR enhance*)) OR TITLE-ABS-KEY (physiotherap* OR ndt OR bobath* OR vojta*)) AND (TITLE-ABS-KEY (motor W/3 (skill* OR ability* OR develop* OR outcome* OR function* OR gross OR fine OR activit* OR performance OR improve*)) OR TITLE-ABS-KEY ((limb OR extremit* OR hand OR foot) W/3 (function OR skill* OR ability*)) OR TITLE-ABS-KEY (outcome OR movement OR gait)) AND ORIG-LOAD-DATE > 20200512

(TITLE-ABS-KEY (((congenital* OR hereditary OR inborn) AND ((heart* OR cardiac* OR coronary OR septal* OR aortopulmonary OR aorticopulmonary OR atrial OR ventricular OR intraventricular) W/3 (defect* OR disease* OR malformation* OR abnormal* OR anomal*))) OR (digeorge W/1 (syndrome* OR anomal* OR sequenc*)) OR (transpos* W/3 (arteries OR artery OR vessel*)) OR (alagille W/2 syndrome) OR ("arteriohepatic dysplasia*" OR "gonadal dysgenesis" OR "subdivided left atrium*") OR ((cardiovertebral OR "pharyngeal pouch" OR "thymic aplasia" OR "conotruncal anomaly face" OR turner* OR noonan OR barth OR velo* OR kartagener* OR siewert* OR scimitar OR lutembacher* OR leopard OR "multiple lentigines" OR marfan*) W/3 syndrome*) OR ("hepatic hypoplasia" OR "arteriohepatic dysplasia*" OR "bicuspid aortic valve") OR (taussig* W/2 anomal*) OR ((pulmon* OR aortic OR subaortic OR valve OR mitral) W/1 stenosis) OR ((aortic OR aorta*) W/3 coarctation*) OR (ventricular W/2 dysplasia*) OR ("cor triatriatum" OR cortriatriatum OR "triatrial heart*") OR ("myocardial bridging*" OR "crisscross heart*" OR "criss-cross heart*") OR (dextrocardia* OR "kartagener* triad" OR "primary ciliary dyskinesia") OR ("patent ductus arteriosus" OR "anomalous pulmonary venous connection" OR "double inlet left ventricle" OR "double outlet right ventricle" OR "interrupted aortic arch") OR ("ebstein* anomaly" OR "ebstein* malformation*" OR
3 "ectopia cordis") OR (eisenmenger* W/1 (complex OR syndrome)) OR ("persistent 2
truncus arteriosus" OR "persistent ostium primum") OR ("endocardial cushion defect*" OR "atrioventricular canal") OR ("foramen oval*") OR (heart W/3 hypoplas*) OR ((noncompaction OR "non compaction") W/3 "ventricular myocardium") OR (levocardia) OR (((tetralogy OR trilogy OR syndrome) W/2 fallot*) OR cantrell* OR shon?s) OR ((tricuspid OR valve OR pulmonary) W/1 atresia*) OR ("absent right atrioventricular connection" OR "single ventricle physiology" OR guch OR "cavopulmonary connection") OR ((bonnevie W/2 (syndrome* OR status)) OR "polynesian bronchiectas*"))) AND (TITLE-ABS-KEY (infant* OR baby OR babies OR neonat* OR newborn OR preterm OR prematur* OR "after birth")) AND (TITLE-ABS-KEY ((physical OR occupational OR constraint-induced OR neurodevelopment* OR neuro-development* OR motor OR movement OR exercise) W/3 (train* OR therap* OR intervention* OR treat* OR support* OR enhance*)) OR TITLE-ABS-KEY (physiotherap* OR ndt OR bobath* OR vojta*)) AND (TITLE-ABS-KEY (motor W/3 (skill* OR ability* OR develop* OR outcome* OR function* OR gross OR fine OR activit* OR performance OR improve*)) OR TITLE-ABS-KEY ((limb OR extremit* OR hand OR foot) W/3 (function OR skill* OR ability*)) OR TITLE-ABS-KEY (outcome OR movement OR gait)) AND (LIMIT-TO (LANGUAGE , "English") OR LIMIT-TO (LANGUAGE , "German") OR LIMIT-TO (LANGUAGE , "French"))

View Less ^

2 (TITLE-ABS-KEY (((congenital* OR hereditary OR inborn) AND ((heart* OR cardiac* OR coronary OR septal* OR aortopulmonary OR aorticopulmonary OR atrial OR ventricular OR intraventricular) W/3 (defect* OR disease* OR malformation* OR abnormal* OR anomal*))) OR (digeorge W/1 (syndrome* OR anomal* OR sequenc*)) OR (transpos* W/3 (arteries OR artery OR vessel*)) OR (alagille W/2 syndrome) OR ("arteriohepatic dysplasia*" OR "gonadal dysgenesis" OR "subdivided left atrium*") OR ((cardiovertebral OR "pharyngeal pouch" OR "thymic aplasia" OR "conotruncal anomaly face" OR turner* OR noonan OR barth OR velo* OR kartagener* OR siewert* OR scimitar OR lutembacher* OR leopard OR "multiple lentigines" OR marfan*) W/3 syndrome*) OR ("hepatic hypoplasia" OR "arteriohepatic dysplasia*" OR "bicuspid aortic valve") OR (taussig* W/2 anomal*) OR ((pulmon* OR aortic OR subaortic OR valve OR mitral) W/1 stenosis) OR ((aortic OR aorta*) W/3 coarctation*) OR (ventricular W/2 dysplasia*) OR ("cor triatriatum" OR cortriatriatum OR "triatrial heart*") OR ("myocardial bridging*" OR "crisscross heart*" OR "criss-cross heart*") OR (dextrocardia* OR "kartagener* triad" OR "primary ciliary dyskinesia") OR ("patent ductus arteriosus" OR "anomalous pulmonary venous connection" OR "double inlet left ventricle" OR "double outlet right ventricle" OR "interrupted aortic arch") OR ("ebstein* anomaly" OR "ebstein* malformation*" OR "ectopia cordis") OR (eisenmenger* W/1 (complex OR syndrome)) OR ("persistent truncus arteriosus" OR "persistent ostium primum") OR ("endocardial cushion defect*" OR "atrioventricular canal") OR ("foramen oval*") OR (heart W/3 hypoplas*) OR ((noncompaction OR "non compaction") W/3 "ventricular myocardium") OR (levocardia) OR (((tetralogy OR trilogy OR syndrome) W/2 fallot*) OR cantrell* OR shon?s) OR ((tricuspid OR valve OR pulmonary) W/1 atresia*) OR ("absent right atrioventricular connection" OR "single ventricle physiology" OR guch OR "cavopulmonary connection") OR ((bonnevie W/2 (syndrome* OR status)) OR "polynesian bronchiectas*"))) AND (TITLE-ABS-KEY (infant* OR baby OR babies OR neonat* OR newborn OR preterm OR prematur* OR "after birth")) AND (TITLE-ABS-KEY ((physical OR occupational OR constraint-induced OR neurodevelopment* OR neuro-development* OR motor OR movement OR exercise) W/3 (train* OR therap* OR intervention* OR treat* OR support* OR enhance*)) OR TITLE-ABS-KEY (physiotherap* OR ndt OR bobath* OR vojta*)) AND (TITLE-ABS-KEY (motor W/3 (skill* OR ability* OR develop* OR outcome* OR function* OR gross OR fine OR activit* OR performance OR improve*)) OR TITLE-ABS-KEY ((limb OR extremit* OR hand OR foot) W/3 (function OR skill* OR ability*)) OR TITLE-ABS-KEY (outcome OR movement OR gait))

View Less
